# Development of a ferroptosis-based model to predict prognosis, tumor microenvironment, and drug response for lung adenocarcinoma with weighted genes co-expression network analysis

**DOI:** 10.3389/fphar.2022.1072589

**Published:** 2022-11-17

**Authors:** Tao Cheng, Guangyao Shan, Huiqin Yang, Jie Gu, Chunlai Lu, Fengkai Xu, Di Ge

**Affiliations:** Department of Thoracic Surgery, Zhongshan Hospital, Fudan University, Shanghai, China

**Keywords:** ferroptosis, WGCNA, lung adenocarcinoma, bioinformatics analysis, prognosis, immune microenvironment, drugs sensitivity

## Abstract

**Objective:** The goal of this study was to create a risk model based on the ferroptosis gene set that affects lung adenocarcinoma (LUAD) patients’ prognosis and to investigate the potential underlying mechanisms.

**Material and Methods:** A cohort of 482 LUAD patients from the TCGA database was used to develop the prognostic model. We picked the module genes from the ferroptosis gene set using weighted genes co-expression network analysis (WGCNA). The least absolute shrinkage and selection operator (LASSO) and univariate cox regression were used to screen the hub genes. Finally, the multivariate Cox analysis constructed a risk prediction score model. Three other cohorts of LUAD patients from the GEO database were included to validate the prediction ability of our model. Furthermore, the differentially expressed genes (DEG), immune infiltration, and drug sensitivity were analyzed.

**Results:** An eight-gene-based prognostic model, including PIR, PEBP1, PPP1R13L, CA9, GLS2, DECR1, OTUB1, and YWHAE, was built. The patients from the TCGA database were classified into the high-RS and low-RS groups. The high-RS group was characterized by poor overall survival (OS) and less immune infiltration. Based on clinical traits, we separated the patients into various subgroups, and RS had remarkable prediction performance in each subgroup. The RS distribution analysis demonstrated that the RS was significantly associated with the stage of the LUAD patients. According to the study of immune cell infiltration in both groups, patients in the high-RS group had a lower abundance of immune cells, and less infiltration was associated with worse survival. Besides, we discovered that the high-RS group might not respond well to immune checkpoint inhibitors when we analyzed the gene expression of immune checkpoints. However, drug sensitivity analysis suggested that high-RS groups were more sensitive to common LUAD agents such as Afatinib, Erlotinib, Gefitinib, and Osimertinib.

**Conclusion:** We constructed a novel and reliable ferroptosis-related model for LUAD patients, which was associated with prognosis, immune cell infiltration, and drug sensitivity, aiming to shed new light on the cancer biology and precision medicine.

## Introduction

Lung cancer, short for primary bronchogenic carcinoma, is the malignant tumor with the highest mortality and morbidity worldwide (Sung et al., 2021). From the point of pathology and therapy, lung cancer can be divided into non-small cell lung cancer (NSCLC) and small cell lung cancer (SCLC). NSCLC counted for 80%–85%, which can be further classified into lung adenocarcinoma (LUAD) and lung squamous cell carcinoma (LUSC) for most patients. LUAD represents the most common lung cancer subtype (Inamura, 2018). Although low-dose computed tomography showed an advantage in lung cancer diagnosis, until recently, no effective lung cancer screening method was available, which resulted in the advanced stage when the patients were diagnosed (Nasim et al., 2019). As scientific research develops, more and more therapeutic modalities are being applied to treat lung cancer. Neoadjuvant radiation and neoadjuvant immunotherapy have recently emerged in addition to traditional surgery. Researchers have demonstrated that Nivolumab is the most effective treatment for patients with advanced NSCLC who have high PD-L1 expression (>50%) (Liang et al., 2020). In addition to traditional chemotherapy, immunotherapy and targeted therapies, studies have shown that root extracts of some plants, such as Plant-Derived Triptolide and Tanshinone I, are beneficial in anti-tumor treatment (Yan et al., 2018; Wei et al., 2019). However, this has not significantly improved the relatively poor prognosis of lung cancer patients. The overall 5-year relative survival rate was less than 30% for NSCLC and only less than 10% for SCLC (Nasim et al., 2019).

Ferroptosis was a novel type of iron-dependent cell death discovered recently, accompanied by massive iron accumulation and lipid peroxidation (Li et al., 2020a). Ferroptosis resulted from the redox imbalance between oxidants and antioxidants, which led to the accumulation of lipid reactive oxygen species (Tang et al., 2021), ultimately causing oxidative cell death. Numerous preclinical studies indicated that the stimulation of ferroptosis might be a helpful therapeutic approach to avoid the development of acquired resistance to a number of cancer treatments (Hangauer et al., 2017; Viswanathan et al., 2017; Tsoi et al., 2018). Regarding immunotherapy, ICIs targeted CTLA4, PD-1, and its ligand PD-L1 and worked primarily by triggering an efficient cytotoxic T cell-driven anti-tumor immune response. Cancer cells might undergo ferroptosis as a result of cytotoxic T-cell-driven immunity (Wang et al., 2019). A complex web of epigenetic, transcriptional, post-transcriptional, and post-translational processes controlled the ferroptotic response. Targeting the mechanisms that control ferroptosis in tumor cells could be a new anticancer tactic (Chen et al., 2021a; Tang et al., 2021).

Currently, it is unclear what mechanisms ferroptosis plays in lung cancer. In this study, bioinformatics methods were used to examine the interactions between genes related to ferroptosis and lung cancer. Subsequently, we analyzed the infiltration of the immune cells in tumor tissue.

## Materials and methods

### Materials and samples

We acquired mRNA-seq expression data, survival statistics, and clinical details for patients with LUAD from the Cancer Genome Atlas (TCGA) data portal (https://portal.gdc.cancer.gov/) (TCGA-LUAD). After matching the mRNA expression and miRNA expression with survival files, mRNA-seq data of 497 patients and miRNA data of 416 patients were utilized for further investigation. Additionally, we recruited GSE8894, GSE50081, and GSE68465 data sets with intact mRNA-seq expression data and survival statistics as validation cohorts by searching the Gene Expression Omnibus (GEO) database.

### Ferroptosis-related genes

FerrDb (Zhou and Bao, 2020) (http://www.datjar.com:40013/bt2104/) was the first database dedicated to ferroptosis regulators and ferroptosis-disease associations. A total of 407 FRGs (255 driven genes, 208 suppressors genes, and 125 marker genes, 131 were overlapped) were extracted (Supplementary Table S1) for subsequent analysis.

### Weighted gene co-expression network analysis

Weighted gene co-expression analysis can systematically detect strongly associated modules in a gene set. WGCNA, as an unsupervised algorithm, can construct a correlation between gene expression and clinical traits (Langfelder and Horvath, 2008). Instead of concentrating simply on differentially expressed genes, WGCNA identifies gene sets of interest and does substantial association analysis with phenotypes, which transforms the problem of multiple hypothesis testing corrections by changing the correlation of thousands of genes with phenotypes into the association of several gene sets. WGCNA contributed to identifying susceptibility modules and genes in multiple diseases and malignant carcinoma, for example, in abdominal aortic aneurysm, NSCLC, and esophageal adenocarcinoma (Chen et al., 2019; Niemira et al., 2019; Nangraj et al., 2020).

With the gene expression of tumor tissues, the Estimation of STromal and Immune cells in MAlignant Tumors using Expression data (ESTIMATE) (Yoshihara et al., 2013) algorithm could predict the proportion of the stromal and immune cells in tumor samples. The results of these tools included immune score, stromal score, and ESTIMATE score, which are positively correlated with the infiltration level of the stromal and immune cells in tumor tissues and tumor purity. We carried out this study using WGCNA with the four results as the phenotypes to analyze the gene expression of LUAD.

### Identification of the hub genes and construction of a ferroptosis-related prognostic model

The module genes screened by WGCNA were analyzed with univariate Cox regression to retrieve prognostic FRGs, using a *p* value <0.05 as the threshold. And then, the least absolute shrinkage and selection operator (LASSO) regression analysis was performed with the glmnet package (Liang et al., 2022). The penalty parameter (λ) value was determined according to the lowest partial likelihood of deviance by 10-fold cross-validation. The genes selected from LASSO regression were the hub genes.

After recognizing the hub genes, we constructed a ferroptosis-related prognostic model with multivariate Cox regression analysis. The risk score (RS) was also generated after multivariate Cox regression. An RS was the sum of the product of coefficients and gene expression for each patient, in which coefficients indicated the regression coefficient in the multivariate Cox regression, and gene expression was the expression of the hub genes.

Subsequently, the LUAD patients in the TCGA cohort were allocated into the high-RS or low-RS group according to the median of the RS. Meanwhile, we created a ferroptosis-related score with the Gene Set Variation analysis (GSVA) to compare the difference in the ferroptosis between the two groups. To evaluate the predictivity of the prognostic model, we performed the Kaplan–Meier (K-M) survival analysis. Furthermore, we used the timeROC package to construct the receiver operating characteristic (ROC) curve to show the one-, two-, and 3-year OS prediction. We determined the discrimination power of RS with the area under the curve (AUC) value. To further explore the reliability of our ferroptosis-related prognostic model, we evaluated the performance of the model in the validation datasets from GEO.

### Evaluation of the prognostic model

We explored the relationship between the RS and different clinicopathologic features among the LUAD patients in the TCGA cohort. Subsequently, we performed the subgroup analysis to further examine if the efficiency of the prognostic model was subject to clinicopathologic characteristics such as gender and TNM stage.

Next, we conducted the univariate and multivariate Cox regression analysis by incorporating RS and the clinical variables, including age, gender, and stage. Based on the regression analysis, a nomogram was constructed to predict the risk of the patients with the rms package. Meanwhile, calibration plots were depicted to assess the prognostic accuracy of the nomogram.

### Analysis of differentially expressed genes, microRNAs, and long-non-coding RNAs

We detected the DEGs with the limma package (Lu et al., 2022) to investigate the difference between the high-RS and low-RS groups. The cutoff criterion for DEGs was a *p* value <0.05 and the absolute value of log2 Fold-change (logFC) > 1. Next, we analyzed the protein-protein interaction (PPI) among the DEGs with the STRING (version 11.5). STRING is an online analysis website that aims to integrate all known and predicted associations between proteins, including physical interactions and functional associations (Szklarczyk et al., 2021). We used the MCODE to visually show the PPI clusters with the interactions of the DEGs. The MODE is a software project most used for visually integrating the protein-protein network in the Cytoscape (Shannon et al., 2003) (version 3.8.2).

Meanwhile, we detected the differentially expressed miRNAs and lncRNAs between the two groups. The criterion for miRNAs was a *p* value <0.05 and the absolute of logFC >0.5. The standard for lncRNAs was a *p* value <0.05 and the absolute of logFC >1.

### Analysis of the tumor microenvironment

In our study, the single-sample Gene Set Enrichment Analysis (ssGSEA) algorithm was performed with the GSVA package to calculate the single-sample enrichment score of 24 immune cell types (Bi et al., 2020). Meanwhile, Spearman correlation analysis was performed to evaluate the relationship between the infiltrating immune cells. The expression of immune checkpoints in different groups was explored for that they were significantly related to the response to immunotherapy (Cai et al., 2021). Furthermore, the potential immune checkpoint blockade (ICB) response was predicted with the tumor immune dysfunction and exclusion (TIDE) algorithm (http://tide.dfci.harvard.edu/) (Fu et al., 2020).

### Prediction of drug sensitivity

We exploited cell line drug sensitivity data in the Genomics of Drug Sensitivity in Cancer (GDSC; https://www.cancerrxgene.org/) database (Yang et al., 2013) in order to identify drugs that LUAD patients might most benefit from. The drug sensitivity was measured with the oncoPredict package by half-maximal drug inhibitory concentration (IC50) (Park et al., 2022).

### Statistical analysis

All the statistical analysis was performed in the R software (version 4.1.3). The Chi-square test or Fisher exact test was used for categorical variables when appropriate and Student’s t-test for continuous variables. The log-rank test was used to compare the overall survival (OS) between the two groups. All the tests were two-sided and the significance threshold for the *p*-value was 0.05.

## Results

### Weighted genes co-expression network analysis and identification of the hub genes

**FIGURE 1:**
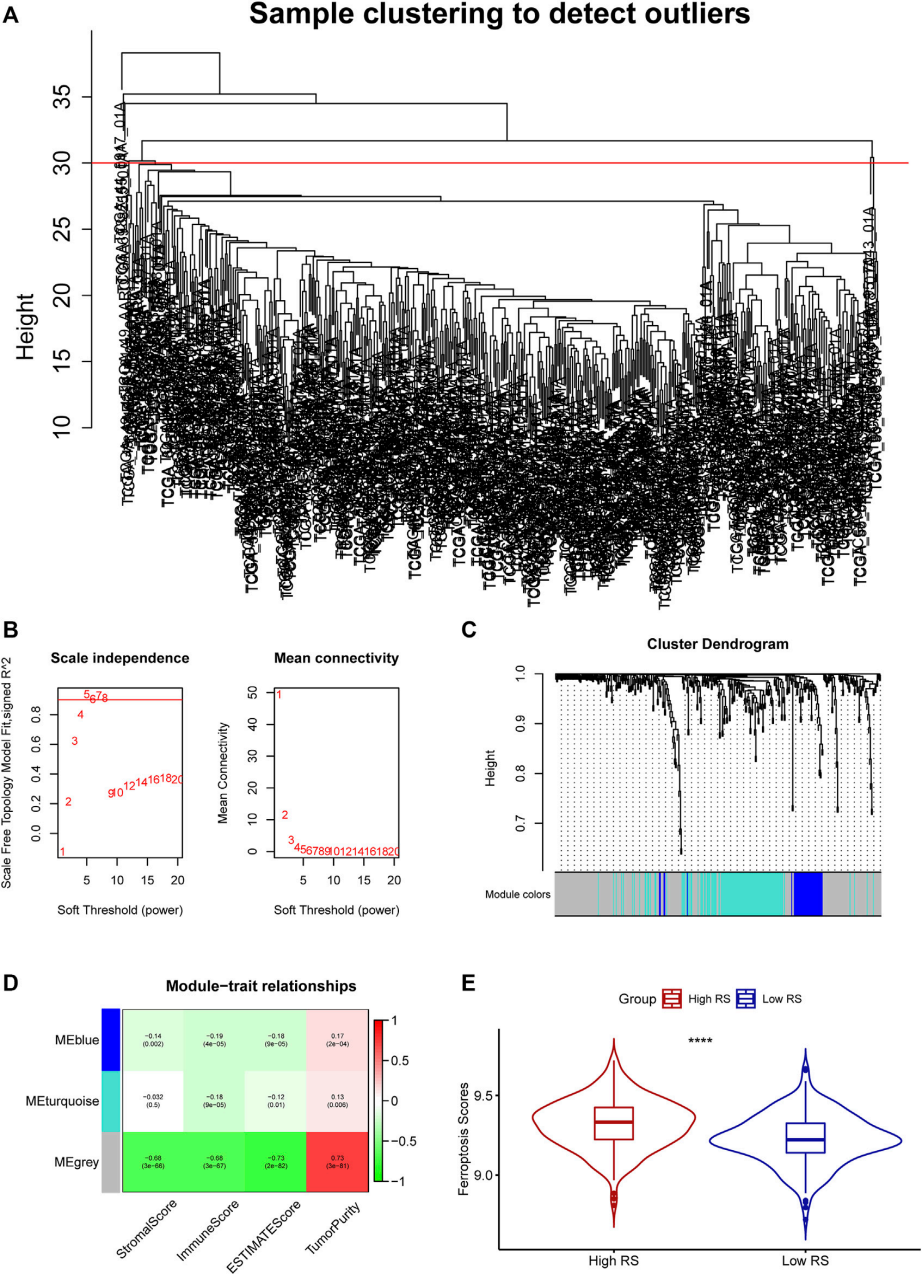
**(A)** Clustering of samples and removal of outliers. **(B)** Analysis of network topology for various soft-thresholding powers. **(C)** The cluster dendrogram of genes of LUAD patients. Each branch in the figure represents one gene, and every color below represents one co-expression module. **(D)** Correlation between the gene module and clinical characteristics, including stromal score, immune score, ESTIMATE score, and tumor purity. **(E)** The ferroptosis-related score difference between the two groups.

With the ESTIMATE algorithm and the RNA-seq expression profile, we obtained the stromal score, immune score, estimate score, and tumor purity of the 497 LUAD patients in the TCGA cohort. After intersecting the RNA-seq expression profile with the FRGs and removing the outlier samples (Figure 1A), 362 FRGs and 482 samples in the TCGA cohorts were incorporated into the co-expression network analysis using the four signatures calculated in the ESTIMATE. The power of *β* = 5 was chosen as the soft-thresholding parameter to ensure a scale-free network (Figure 1B). The 362 FRGs were separated into three modules named blue, turquoise, and grey, among which the grey module (*R* ^2^ = 0.68, *p* < 0.0001) was significantly correlated to the immune score (Figures 1C,D).

**FIGURE 2:**
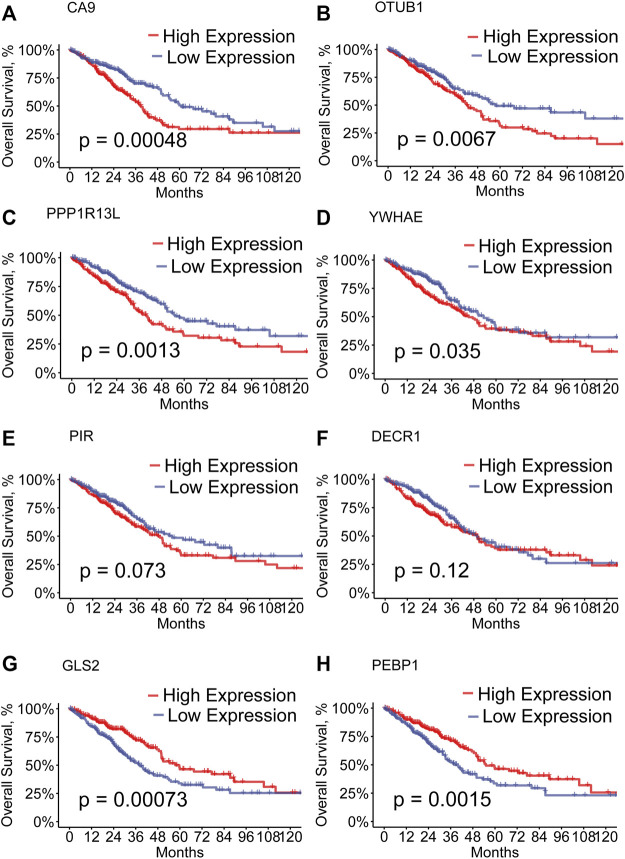
**(A–H)** Kaplan–Meier curves of high- and low-expression of the hub genes of LUAD patients in the TCGA.

We performed the univariate Cox regression analysis with the 207 module genes in the grey module and selected 31 prognostic genes (*p* < 0.05). To remediate multicollinearity among these genes, we performed the LASSO analysis (Supplementary Figures S1A,B) and eight hub genes, including PIR, PEBP1, PPP1R13L, CA9, GLS2, DECR1, OTUB1, and YWHAE, were finally included in the prognostic model. Among these eight hub genes, GLS2 and PEBP1 were downregulated in the high-RS group, and the remaining ones were upregulated. In addition, we performed K-M survival analysis of each hub gene. We found that patients with the high expression of PPP1R13L, CA9, OTUB1, and YWHAE and low expression of GLS2 and PEBP1presented a worse OS (Figures 2A–D,G,H, *p* < 0.05). As mentioned previously, GLS2 and PEBPB1 were downregulated and the other ones were upregulated in the high-RS group, which similarly showed a worse OS. However, survival differences were not detected for PIR and DECR1. (Figures 2E,F).

### Construction of the ferroptosis-related prognostic model

**FIGURE 3:**
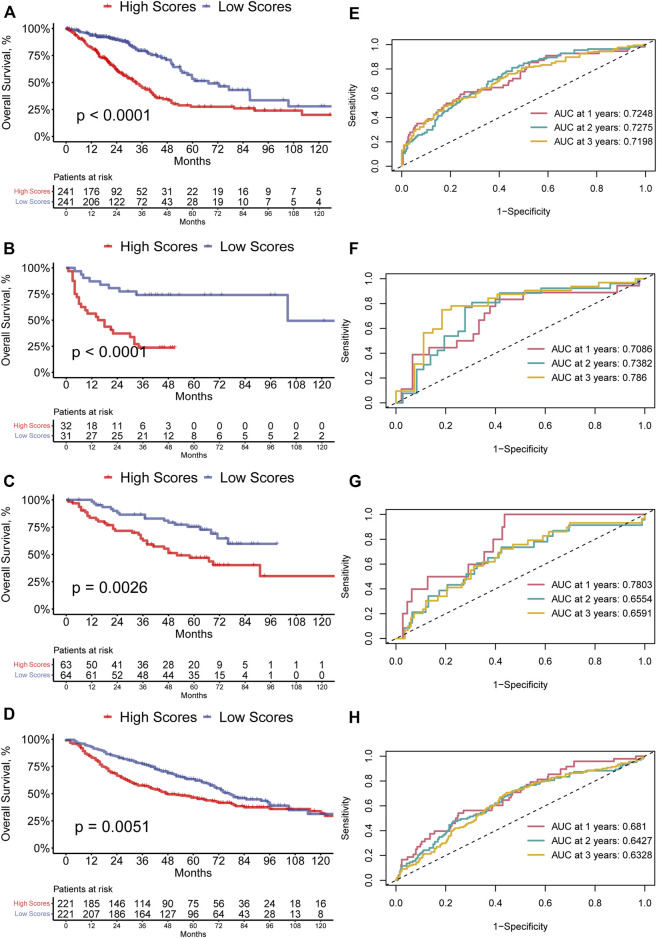
**(A–D)** Kaplan–Meier curves of high- and low-RS LUAD patients in the TCGA, GSE8894, GSE50081, and GSE68465 cohort. **(E–H)** ROC curves of one-, two-, and 3-year OS for LUAD patients based on the RS in the TCGA, GSE8894, GSE50081, and GSE68465 cohort.

The ferroptosis-related prognostic model for LUAD patients was built with the multivariate Cox regression analysis and the RS was calculated as followed: PIR * (0.23113668) + PEBP1 * (−0.54954902) + PPP1R13L * (0.23748274) + CA9 * (0.07352803) **+** GLS2 * (−0.50852672) + DECR1 * (0.33163909) + OTUB1 * (0.34034368) + YWHAE * (0.38633985). Then the LUAD patients were divided into the high-(*N* = 241) and low-RS (*N* = 241) groups by the median RS. The GSVA showed that the high-RS group enriched a higher ferroptosis-related score (*p* < 0.05, Figure 1E). Furthermore, the K-M survival analysis demonstrated that the high-RS group had a worse OS (*p* < 0.05, Figure 3A), indicating that high RS might be a high risk for LUAD patients. The predictive ability of our model was visualized by the ROC curve and quantified by the AUC. The result showed the AUC at one-, two-, and 3-year OS prediction was 0.7248, 0.7275, and 0.7198, respectively (Figure 3E).

### External validation of the ferroptosis-related prognostic model

Additionally, the predictive ability of our model was validated in the dataset from GEO. The high-RS group in GSE8894 also had a worse survival (*p* < 0.0001, Figure 3B), and the AUC at one-, two-, and 3-year OS prediction was 0.7086, 0.7382, and 0.786 (Figure 3F). Similarly, in the GSE50081 and the GSE68465 dataset, the K-M survival analysis implied the same tendency (Figures 3C,D). The AUC at one-, two-, and 3-year OS prediction was also greater than 0.6 (Figures 3G,H). These results suggested that the ferroptosis-related prognostic model showed a robust prognostic ability.

### Clinical signature of the model

**FIGURE 4:**
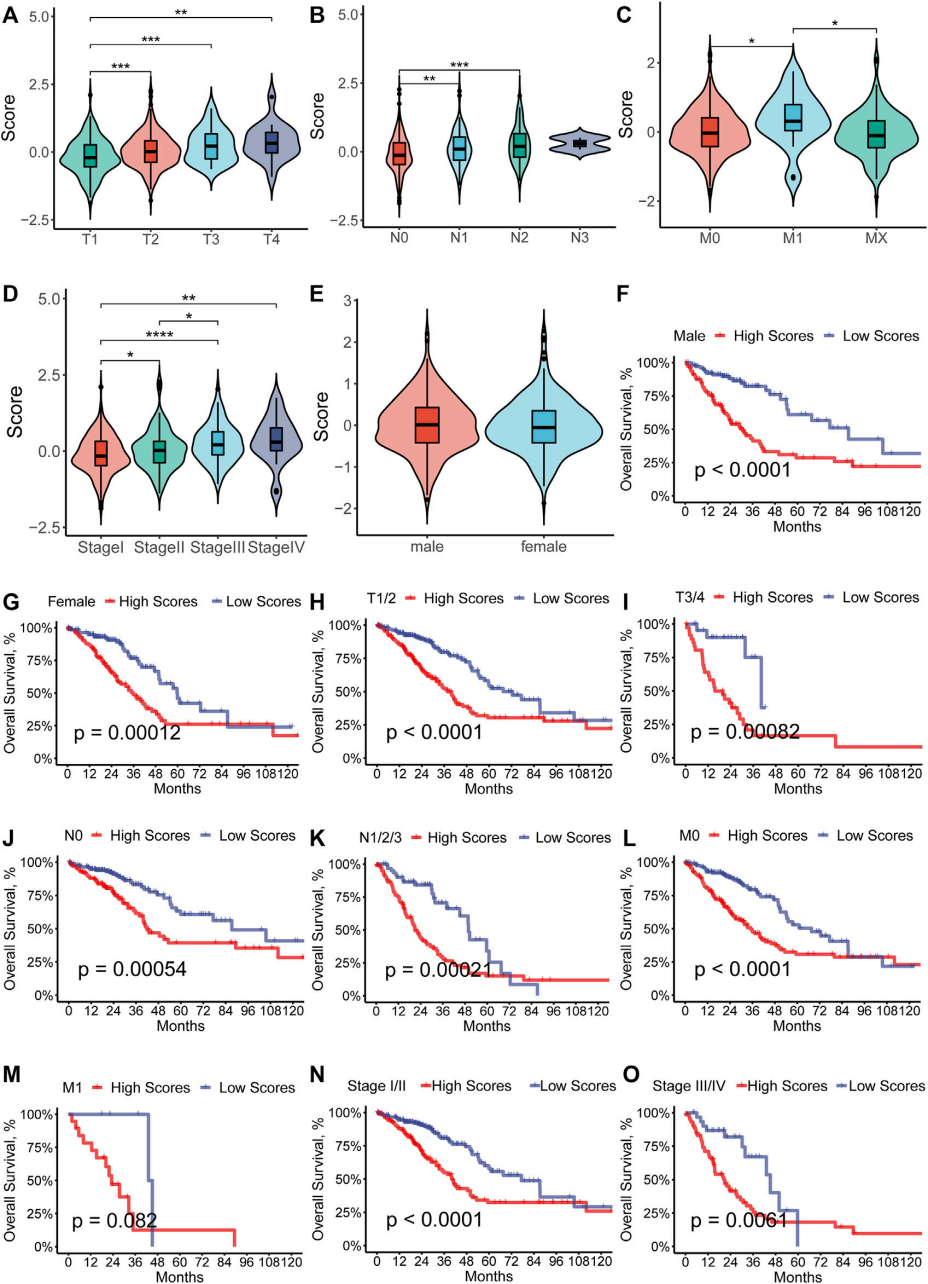
**(A–E)** Distribution of the RS separated by the clinical-pathological features among the LUAD patients in the TCGA cohort. **(F–O)** Subgroup analysis of prognostic value of the ferroptosis-prognostic model for LUAD patients by Kaplan–Meier curves according to clinicopathologic characteristics.

To further investigate the relationship between the RS and the different clinical characteristics, we compared the distribution of the RS in the subgroup of LUAD patients. The results suggested that the RS increased as the TNM stage advanced (*p* < 0.05, Figures 4A–D), which demonstrated that the RS implied the progression of LUAD. There was no significant difference in the distribution of the RS in different gender (Figure 4E). For further analysis of the predictive ability of the RS, we performed the survival analysis in the subgroup. From the result, we could see that the higher-RS group showed worse OS than the low-RS group in almost every subgroup (Figures 4F–O) except for the M1 group. It is plausible that the insufficient sample number (*N* = 24) for the M1 subgroup was the cause of the lack of survival differences.

**FIGURE 5:**
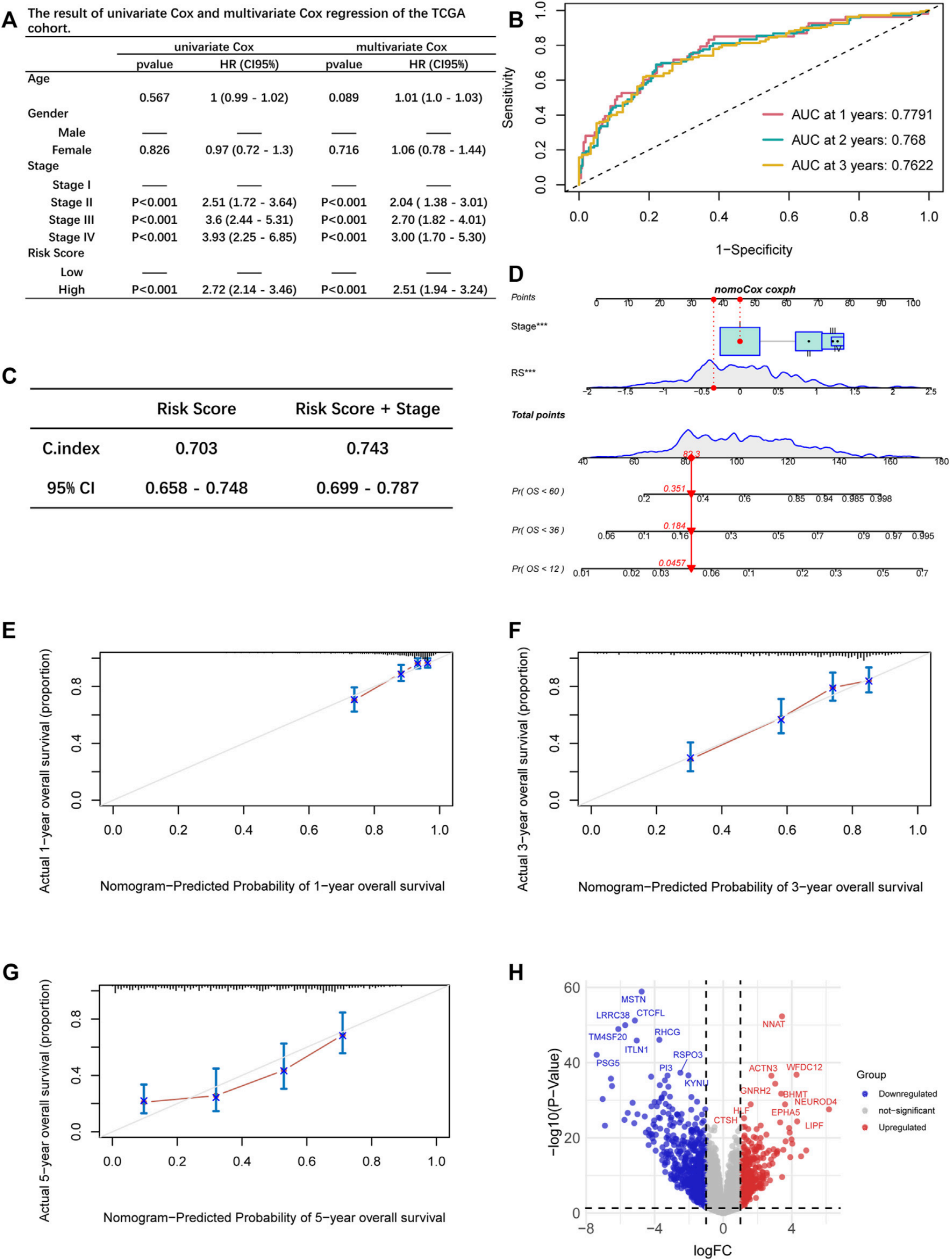
**(A)** Uni- and multivariate Cox regression analysis of the associations between survival outcomes and age, gender, stage, and risk score of LUAD patients. **(B)** ROC curves of one-, two-, and 3-year OS for LUAD patients based on the RS and TNM stage. **(C)** The comparison of the prediction ability between the two model. **(D)** The nomogram of the overall survival prediction model. **(E–G)** Calibration plots for the nomogram: 1-year **(E)**; 3-year **(F)**; 5-year **(G)** nomogram. **(H)** Volcano plot of differentially expressed genes between the two groups.

We performed the univariate and multivariate Cox regression by incorporating the RS and the clinical characteristics, including age, gender, and stage, demonstrating that tumor stage and RS were the independent risk factors of LUAD (*p* < 0.05, Figure 5A). The AUC at one-, two-, and 3-year OS prediction was 0.7791, 0.768, and 0.7622, separately (Figure 5B). Meanwhile, the corresponding concordance index (C-index) value showed that the combination of the RS and stage performed remarkably (Figure 5C). A nomogram was constructed according to independent risk factors to predict the risk of the patients (Figure 5D). The calibration plots were depicted to show the one-, three-, and 5-year OS rates of LUAD patients (Figures 5E–G).

### Analysis of the differentially expressed genes

**FIGURE 6:**
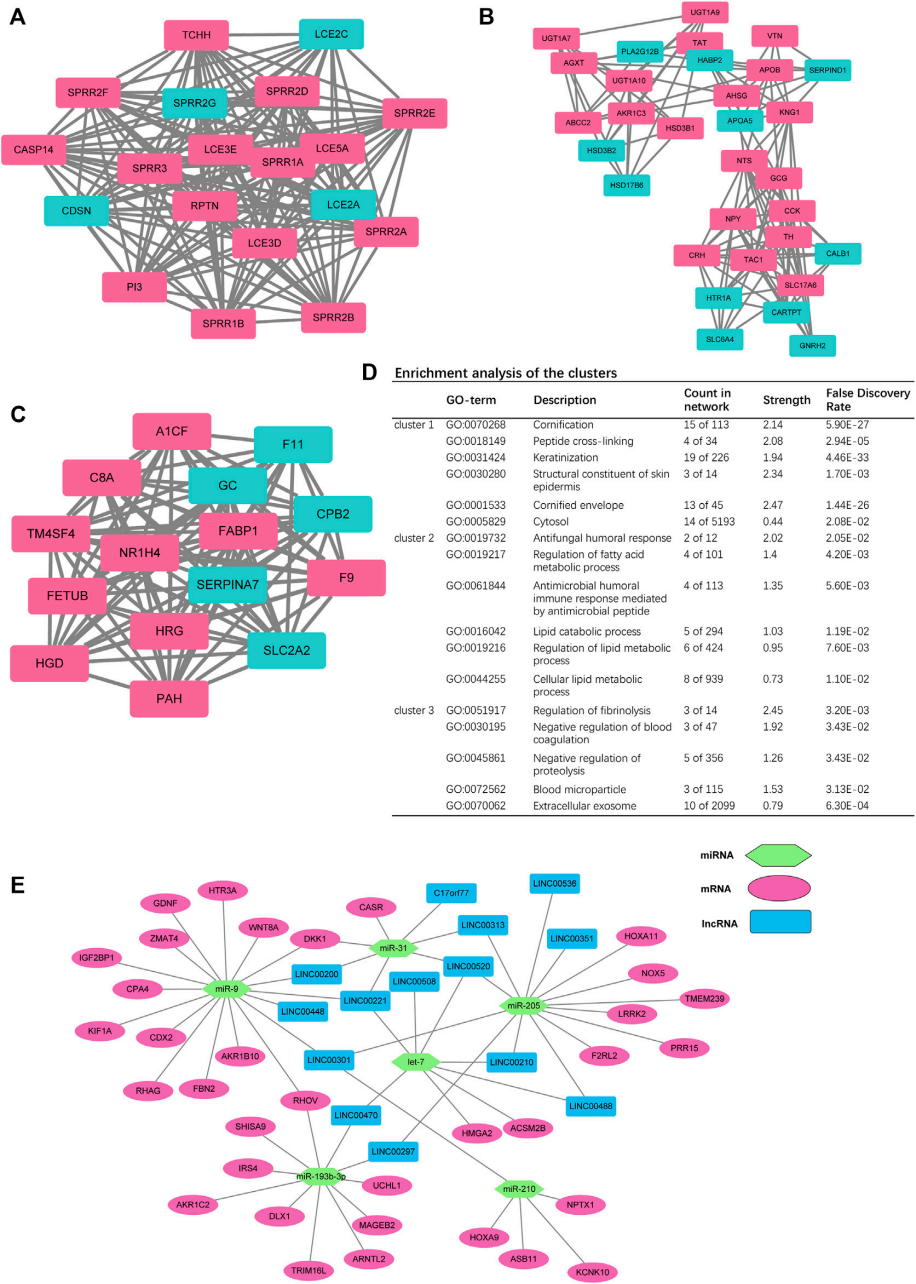
**(A–C)** Top three clusters in the protein-to-protein interaction (PPI) network. **(D)** Enrichment analysis of the top three clusters. **(E)** Competing endogenous RNA (ceRNA) network of RS-related DEGs - differentially expressed miRNAs—differentially expressed lncRNAs.

Among the 18437 protein-coding genes, 380 were upregulated, and 642 were downregulated in the high-RS group (*p* < 0.05, Figure 5H, Supplementary Table S2). Among the DEGs, NEUROD4 (logFC = 6.166719, *p* < 0.0001), SPAG11B (logFC = 4.839105, *p* < 0.0001), and SPAG11A (logFC = 4.537401, *p* < 0.0001) were the most significantly upregulated genes; PSG5 (logFC = −7.380016, *p* < 0.0001), PSG11 (logFC = −7.033551, *p* < 0.0001), and DEFA5 (logFC = −6.899311, *p* < 0.0001) were the most significantly downregulated genes. Next, the PPI clusters were analyzed with MCODE, and the clustering scores of the top three modules were 19.000, 16.429, and 8.200, respectively (Figures 6A–C). Furthermore, we employed the function enrichment analysis. Lipid metabolism was the most enriched (Figure 6D).

### Differentially expressed miRNAs and lncRNAs and construction of the ceRNA network

The lncRNAs and miRNAs regulated the expressions of various mRNAs to influence tumor progression (Seo et al., 2020). Hence, the expression of miRNAs and lncRNAs were analyzed between the high- and low-RS group (Supplementary Figures S1C, S1D). The results showed that 22 miRNAs were dysregulated in the high-RS group, including 18 upregulated and 4 downregulated ones. For lncRNAs, TUNAR (logFC = 3.896800, *p* < 0.0001) was the most significantly upregulated one among the 133 upregulated lncRNAs and LINC01477 (logFC = −4.0848511, *p* < 0.0001) was the most significantly different one among the 95 downregulated lncRNAs.

The expressions of the lncRNAs and miRNAs were negatively correlated in the ceRNA network (Seo et al., 2020). According to the target pairs of miRNA–mRNA and miRNA–lncRNA, we constructed a ceRNA network (Figure 6E). We found that miR-9 was located at the center of the network.

### Characteristics of tumor microenvironment

**FIGURE 7:**
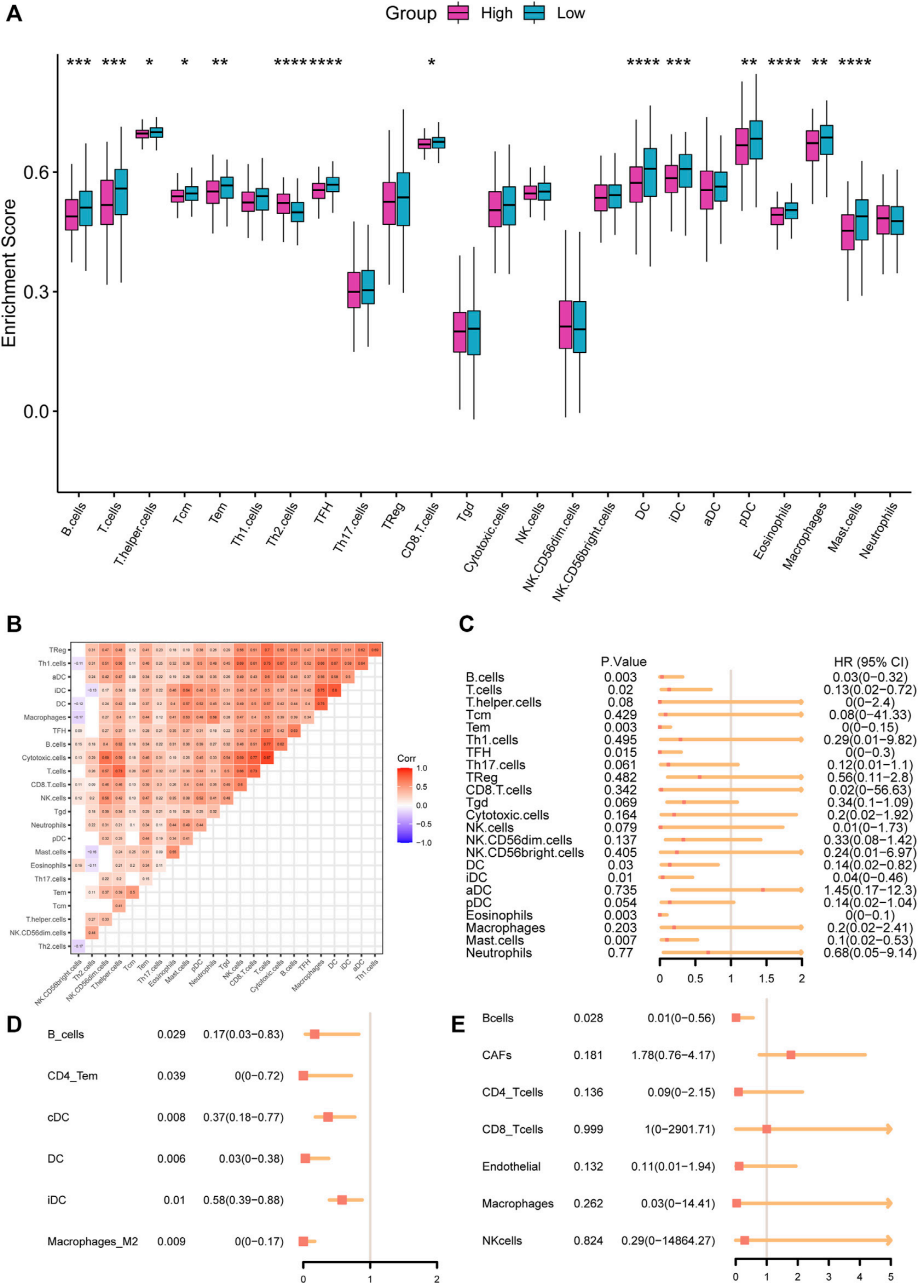
**(A)** Comparisons of infiltration levels of immune cells between high- and low-RS groups with the ssGSEA algorithm. **(B)** The correlation of the tumor-infiltrated immune cells. **(C–E)** Forest plot of the tumor-infiltrated immune cells with ssGSEA **(C)**, xCell database **(D)**, and EPIC database **(E)**.

Immune cells infiltrated in the tumor tissues played an essential role in the TME and tumor progression (Wang et al., 2021). Our research explored the enrichment score of 24 immune cell types to assess the relationship between immune cell infiltration and RS (Figure 7A). The result suggested 14 cell types with lower enrichment scores in the high-RS group, including dendritic cells, B cells, and eosinophils (*p* < 0.05), and Th2 cells with higher enrichment scores in the high-RS group (*p* < 0.001). We further investigated the interrelationships between the 24 immune cell types, which revealed a strong, positive correlation between them (Figure 7B). Last, we performed the univariate Cox regression analysis, which demonstrated that low immune cell infiltration, especially B cells, was related to a poor prognostic (Figures 7C–E).

**FIGURE 8:**
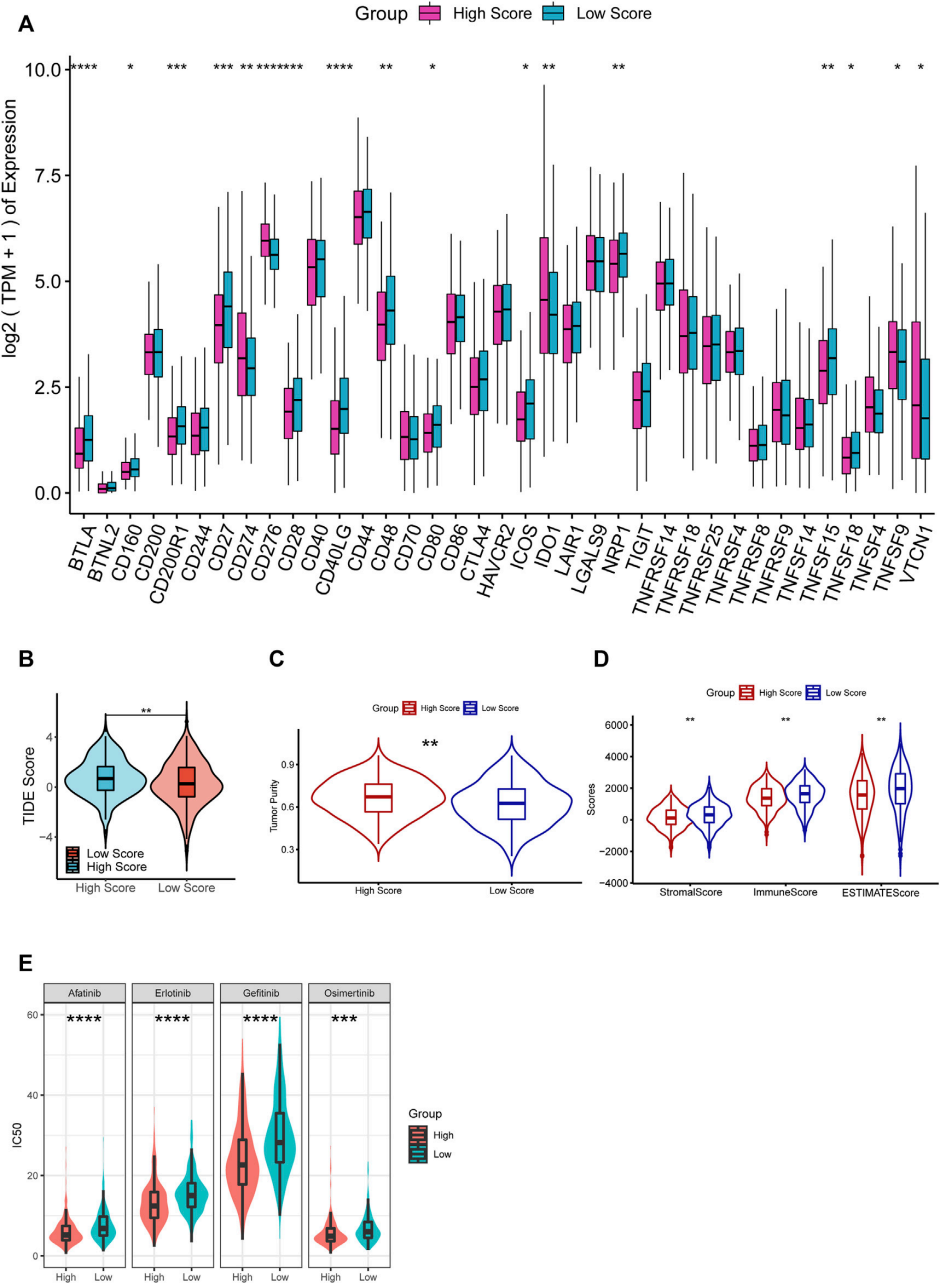
**(A)** Comparisons of the expression of immune checkpoints between high- and low-RS groups. **(B–D)** Comparisons of TIDE score **(B)**, tumor purity **(C)**, stromal score, immune score, and ESTIMATE score **(D)** between the high- and low-RS groups. **(E)** Comparisons of the response to drugs between high- and low-RS groups by GDSC database.

The investigation of the immune checkpoint gene expression suggested that 17 genes are significantly differentially expressed among the 37 immune checkpoint ones, including 12 downregulated and 5 upregulated in the high-RS group (Figure 8A). Moreover, we estimated the potential ICB response with the TIDE algorithm (Figure 8B). The high-RS group showed a higher TIDE score, which indicated a higher potential for tumor immune evasion and a low probability to benefit from anti-PD1/CTLA4 treatment.

With the ESTIMATE algorithm, we compared the Immune Score, Stromal Score, ESTIMATE Score, and tumor purity between the two groups. The results demonstrated that immune, stromal, and Estimate scores were significantly lower, and tumor purity was substantially higher in the high-RS group (*p* < 0.05, Figures 8C,D). These results suggested that high-RS was an indicator of lower immune infiltration, higher tumor purity, and resistance to immunotherapy.

### Prediction of drug sensitivity

We discovered that the response to 90 of the 198 drugs in the GDCS differed considerably between the two groups. Additionally, we observed that the high-RS group was more were more sensitive to 63 of them (Supplementary Table S3). For example, among the agents which were widely used for LUAD patients, including Afatinib, Erlotinib, Gefitinib, and Osimertinib, the result showed that the patients in the high-RS group were more sensitive to them (*p* < 0.001, Figure 8E), which suggested that high-RS might lead to a better response to drugs, and more drugs might be used for LUAD treatment.

## Discussion

LUAD is one of the most prevalent and lethal tumors in adults. This disease imposes a significant financial and medical burden yearly, especially for advanced LUAD (Chen et al., 2021b). In this study, we picked a grey module of 207 genes significantly related to the immune score and ferroptosis by WGCNA. After that, through univariate Cox regression and LASSO analysis, we reduced the gene dimension considerably and screened 8 hub genes, including PIR, PEBP1, PPP1R13L, CA9, GLS2, DECR1, OTUB1, and YWHAE, which were significantly related to the survival of LUAD patients. Finally, we constructed a ferroptosis-related prognostic model, and the LUAD patients were divided into high- and low-risk groups by the RS. In our research, the two groups showed different immune cell infiltration, immune checkpoint expression, and response to drugs.

From the previous research, the hub genes in our model were considered to promote or suppress tumorigenesis. PIR is one of the cupin superfamily memberships acting as a nuclear redox sensor and regulator (Wendler et al., 1997). PIR played a negative role in regulating ferroptosis in multiple cancer cells. The knockdown of PIR increased mRNA levels of ACSL4, a biomarker and a key promoter of ferroptosis, in pancreatic ductal adenocarcinoma cell lines (Hu et al., 2021). PPP1R13L, also known as iASPP, is one of the most evolutionarily conserved inhibitors of p53, which played a central role in the regulation of apoptosis and transcription *via* its interaction with NF-kappa-B and p53/TP53 proteins, therefore suppressing the subsequent activation of apoptosis (Laska et al., 2009). Furthermore, researchers have demonstrated that PPP1R13L increased chemotherapeutic drug resistance in tumor cells *via* the NF-κBp65- and p53-signaling pathways (Li et al., 2020b). Carbonic anhydrases (CAs) are a large family of zinc metalloenzymes that catalyze the reversible hydration of carbon dioxide (Supuran, 2018). They participate in a variety of biological processes. CA9, as a member of the CAs family, has become a biomarker for the therapy of a wide range of cancers (Supuran, 2008). It was reported that CA9 was associated with the migration and invasion of breast cancer cells (Swayampakula et al., 2017) and cervical cancer cells (Shin et al., 2011). In an analysis of 98 tissue samples of NSCLC, patients with high CA9 expression had significantly worse survival than all other groups (Giatromanolaki et al., 2020). Tumor cells are characterized by cellular metabolism abnormalities, including lipid metabolism disorder. DECR1 is an accessory enzyme that participates in the beta-oxidation and metabolism of unsaturated fatty enoyl-CoA esters. As an androgen receptor (AR) target gene with negative regulatory activity, DECR1 might support human prostate cancer (PCa) cell survival and resistance to AR targeting therapies (Nassar et al., 2020). DECR1 knockdown made PCa cells susceptible to ferroptosis and inhibited the formation of PCa cells (Blomme et al., 2020). OTUB1 is the founding member of the ovarian tumor (OTU) domain family of deubiquitinases (DUBs) and is expressed in various tissues in humans (Borodovsky et al., 2002). OTUB1 was essential in respiratory control, adult lung tissue homeostasis, embryogenesis, and cell proliferation (Ruiz-Serrano et al., 2021). Through the suppression of RAS ubiquitination in NSCLC, OTUB1 caused the activation of the MAPK pathway, contributing to the advancement of NSCLC (Baietti et al., 2016). YWHAE belongs to the 14-3-3 family of proteins, which mediate signal transduction by binding to phosphoserine-containing proteins. YWHAE was upregulated in breast cancer cells and patients with overexpressed YWHAE showed a poor survival (Yang et al., 2019).

As for the downregulated genes, PEBP1 encoded a member of the phosphatidylethanolamine-binding family of proteins (Yang et al., 2018). It was reported that PEBP1 dissociated the Raf1-MEK complex and acted as an inhibitor of the Raf1/MEK/ERK pathway by binding to Raf1 (Yeung et al., 2000). PEBP1 was found to be downregulated in several tumor cells and act as a metastasis suppressor (Fu et al., 2003; Hagan et al., 2005). GLS2 is a mitochondrial phosphate-activated glutaminase. Glutamine metabolism is a widely-known target for slowing cancer development, while the p53-inducible gene GLS2 was linked to a unique metabolic role in suppressing tumor growth (Suzuki et al., 2010). According to previous studies, GLS2 expression was decreased in human hepatocellular carcinoma (HCC) due to hypermethylation. Furthermore, *via* negatively regulating the PI3K/AKT pathway, GLS2 was crucial in the tumor suppression of HCC (Liu et al., 2014).

As is well known, tumor-infiltration immune cells are the indication of the response to tumor antigens,and strong immune responses to malignancies have resulted in better clinical outcome (Kotsakis et al., 2016). According to a previous study (Zuo et al., 2020), LUAD patients with higher infiltration of 12 immune cell types had a better prognosis. Only a fewer infiltration of Type 2 T helper cells (Th2) demonstrated a worse OS, which was compatible with our findings. The immune cells enrichment analysis in our prognostic model revealed that lower enrichment scores in the high-RS group, who had a shorter survival time. Furthermore, along with the GSVA, the xCell and the EPIC algorithm, the univariate Cox regression analysis found that the abundance of B cells was significantly associated with the prognosis of LUAD patients, and B cells were the protective factor (*p* < 0.05, HR < 0.5). Tumor-infiltrated B cells could be observed in all stages of lung cancer development (Dieu-Nosjean et al., 2014). B cell was one of the most significant participants in humoral immunity. Tumor-infiltrating B cells in lung cancer could develop into plasma cells and secrete antibodies (Germain et al., 2014). Additionally, accumulating researches indicating that tumor-infiltrated B cells and plasma cells were correlated with better OS (Nielsen and Nelson, 2012; Lohr et al., 2013; Ni et al., 2021), which suggested that B cells exerted an anti-tumor function in tumor immunity. Additionally, B cells could promote T cell responses. It has also been demonstrated that lung cancer patients with highly infiltrating T and B cells nearby live longer (Kinoshita et al., 2016). More research proved that neoadjuvant therapy enhanced anti-tumor immunity by recruiting B cells in NSCLC (Gaudreau et al., 2021). In conclusion, our investigation showed a group of LUAD patients with few infiltrating immune cells and shorter OS. At the same time, several TME deconvolution algorithms indicated that the infiltration of B cells was positively correlated with prognosis.

In our analysis of immune checkpoint molecules expression, we detected the differentially expressed ones. Among them, the top three downregulated genes in the high-RS group were BTLA, CD27, and CD28. BTLA, as a member of the CD28 superfamily, was found to be expressed in tumor-infiltrating lymphocytes (Ning et al., 2021). In addition, decreased BTLA levels predicted poor OS in colorectal cancers (Song and Wu, 2020). CD27 is a co-stimulatory immune-checkpoint receptor. It was reported that augmenting CD27 co-stimulation may assist in anti-tumor immunity (Grant et al., 2017). Among the upregulated immune checkpoint molecules, CD274, much more known as PD-L1, and CD276 were members of the B7 superfamily, through which cancer cells exhibit immune escape (Gou et al., 2020; Liu et al., 2021). Similarly, we discovered that the high-RS of LUAD patients less likely to benefit from ICI treatment based on the higher TIDE score in the high-RS group.

## Conclusion

We constructed a novel and reliable ferroptosis-related model for LUAD patients, which was associated with prognosis, immune cell infiltration, and drug sensitivity, aiming to shed new light on the cancer biology and precision medicine.

## Data Availability

Publicly available datasets were analyzed in this study. This data can be found here: GEO (https://www.ncbi.nlm.nih.gov/geo/), GTEx (https://www.gtexportal.org/home/index.html) and TCGA data portal (https://portal.gdc.cancer.gov/).
