# Supplementary material for: Development of a ferroptosis-based model to predict prognosis, tumor microenvironment, and drug response for lung adenocarcinoma with weighted genes co-expression network analysis
Source: Front Pharmacol. 2022 Nov 17;13:1072589. doi: 10.3389/fphar.2022.1072589 (PMC9712758; doi:10.3389/fphar.2022.1072589)
Supplement: Supplementary file 2 [file Table1.DOCX]

setwd("D:/AaPaper/LUADFerro/")

#####数据读入#####

LUAD_TPM <- read.csv("D:/AaPaper/Date整理/TCGA/LUAD/LUAD_TPM_Tumor.csv")

library(tidyverse)

LUAD_TPM <- LUAD_TPM %>% remove_rownames() %>% column_to_rownames("X")

LUAD_Survival <- read.csv("D:/AaPaper/Date整理/TCGA/LUAD/LUAD_Survival.csv")

LUAD_Survival$X <- NULL

LUAD_Survival$OS.time <- round(LUAD_Survival$OS.time/30,1)

LUAD_Clinical <- read.csv("D:/AaPaper/Date整理/TCGA/LUAD/LUAD_PhenoType.csv")

LUAD_Clinical <- tibble::column_to_rownames(LUAD_Clinical,"X")

#通路基因集

Ferroptosis <- read.csv("D:/AaPaper/LUSC/数据/整理/Ferroptosis Gene Set.csv")

#####评分机制#####

library(IOBR)

scoreIPS <- deconvo_ips(eset = 2^LUAD_TPM-1,project = "TCGA-LUAD",plot=F)

scoreESTIMATE <- deconvo_tme(eset = 2^LUAD_TPM-1,method = "estimate",

platform ="affymetrix")#数据必须non-log scale

#Score <- merge(dplyr::select(scoreESTIMATE,1,2,3,5),dplyr::select(scoreIPS,1,9),by="ID")

Score <- scoreESTIMATE

#####WGCNA#####

# Load the WGCNA package

library(flashClust)

library(iterators)

library(WGCNA);

# The following setting is important, do not omit.

options(stringsAsFactors = FALSE)

#Read in the cancer data set

RawData <- LUAD_TPM[which(rownames(LUAD_TPM) %in% Ferroptosis$Ferroptosis),]

any(duplicated(rownames(RawData))) #检测有无重复的名字

datExpr0 = as.data.frame(t(RawData))

gsg = goodSamplesGenes(datExpr0, verbose = 3);#检测缺失值

gsg$allOK #结果为TRUE，则所有选定基因都用于后续WGCNA

#如果gsg$allOK结果为FALSE，则后续选择good gene用于WGCNA

if (!gsg$allOK)

{

# Optionally, print the gene and sample names that were removed:

if (sum(!gsg$goodGenes) > 0)

printFlush(paste("Removing genes:", paste(names(datExpr0)[!gsg$goodGenes], collapse = ", ")));

if (sum(!gsg$goodSamples) > 0)

printFlush(paste("Removing samples:", paste(rownames(datExpr0)[!gsg$goodSamples], collapse = ", ")));

# Remove the offending genes and samples from the data:

datExpr0 = datExpr0[gsg$goodSamples, gsg$goodGenes]

}

#Ferroptosis结果Removing genes:SNORA16A,XBP1

#聚类

sampleTree = hclust(dist(datExpr0), method = "average");

# Plot the sample tree: Open a graphic output window of size 12 by 9 inches

# The user should change the dimensions if the window is too large or too small.

sizeGrWindow(14,9)

#pdf(file = "Plot/sampleClustering.pdf", width = 14, height = 9);

par(cex = 0.6);

par(mar = c(0,4,2,0))

#dev.off()

plot(sampleTree, main = "Sample clustering to detect outliers", sub="", xlab="", cex.lab = 1.5,

cex.axis = 1.5, cex.main = 2)

# Plot a line to show the cut

abline(h = 30, col = "red");

# Determine cluster under the line

clust = cutreeStatic(sampleTree, cutHeight = 30, minSize = 10)

table(clust)

# clust 1 contains the samples we want to keep.

keepSamples = (clust==1)

datExpr = datExpr0[keepSamples, ]

nGenes = ncol(datExpr)

nSamples = nrow(datExpr)

#clinical traits

datTraits <- Score %>% column_to_rownames("ID")

datTraits <- datTraits[which(rownames(datTraits) %in% rownames(datExpr)),]

collectGarbage() #释放内存？

#重新聚类，包含clinical traits

# Re-cluster samples

sampleTree2 = hclust(dist(datExpr), method = "average")

# Convert traits to a color representation: white means low, red means high, grey means missing entry

traitColors = numbers2colors(datTraits, signed = FALSE);

# Plot the sample dendrogram and the colors underneath.

plotDendroAndColors(sampleTree2, traitColors,

groupLabels = names(datTraits),

main = "Sample dendrogram and trait heatmap")

# Choose a set of soft-thresholding powers

powers = c(c(1:10), seq(from = 12, to=20, by=2))

# Call the network topology analysis function

sft = pickSoftThreshold(datExpr, powerVector = powers, verbose = 5)

# Plot the results:

sizeGrWindow(9, 5)

par(mfrow = c(1,2));

cex1 = 0.9;

# Scale-free topology fit index as a function of the soft-thresholding power

plot(sft$fitIndices[,1], -sign(sft$fitIndices[,3])*sft$fitIndices[,2],

xlab="Soft Threshold (power)",ylab="Scale Free Topology Model Fit,signed R^2",type="n",

main = paste("Scale independence"));

text(sft$fitIndices[,1], -sign(sft$fitIndices[,3])*sft$fitIndices[,2],

labels=powers,cex=cex1,col="red");

# this line corresponds to using an R^2 cut-off of h

abline(h=0.90,col="red")

# Mean connectivity as a function of the soft-thresholding power

plot(sft$fitIndices[,1], sft$fitIndices[,5],

xlab="Soft Threshold (power)",ylab="Mean Connectivity", type="n",

main = paste("Mean connectivity"))

text(sft$fitIndices[,1], sft$fitIndices[,5], labels=powers, cex=cex1,col="red")

# sft$powerEstimate是推荐的软阈值

net = blockwiseModules(datExpr, power = sft$powerEstimate,

TOMType = "unsigned", minModuleSize = 30,

reassignThreshold = 0, mergeCutHeight = 0.25,

numericLabels = TRUE, pamRespectsDendro = FALSE,

saveTOMs = F,verbose = 3)

#open a graphics window

sizeGrWindow(12, 9)

# Convert labels to colors for plotting

mergedColors = labels2colors(net$colors)

# Plot the dendrogram and the module colors underneath

plotDendroAndColors(net$dendrograms[[1]], mergedColors[net$blockGenes[[1]]],

"Module colors",

dendroLabels = FALSE, hang = 0.03,

addGuide = TRUE, guideHang = 0.05)

moduleLabels = net$colors

moduleColors = labels2colors(net$colors)

MEs = net$MEs

geneTree = net$dendrograms[[1]]

# Recalculate MEs with color labels

MEs0 = moduleEigengenes(datExpr, moduleColors)$eigengenes

MEs = orderMEs(MEs0)

moduleTraitCor = cor(MEs, datTraits, use = "p")

moduleTraitPvalue = corPvalueStudent(moduleTraitCor, nSamples)

#heatmap

sizeGrWindow(10,6)

# Will display correlations and their p-values

textMatrix = paste(signif(moduleTraitCor, 2), "\n(",

signif(moduleTraitPvalue, 1), ")", sep = "");

dim(textMatrix) = dim(moduleTraitCor)

par(mar = c(6, 8.5, 3, 3));

# Display the correlation values within a heatmap plot

labeledHeatmap(Matrix = moduleTraitCor,

xLabels = names(datTraits),

yLabels = names(MEs),

ySymbols = names(MEs),

colorLabels = FALSE,

colors = greenWhiteRed(50),

textMatrix = textMatrix,

setStdMargins = FALSE,

cex.text = 0.5,

zlim = c(-1,1),

main = paste("Module-trait relationships"))

# 各基因模块的名字（颜色）

modNames = substring(names(MEs), 3)

#提取感兴趣的module内所有的基因名称

module <- "grey"

moduleGenes <- moduleColors== module

table(moduleGenes)

grey_module <- as.data.frame(dimnames(data.frame(datExpr))[[2]][moduleGenes])

names(grey_module) <- "genename"

write.csv(grey_module,file = "Data/moduleGenes.csv")

#####筛选hubgene没结果####

#筛选hub gene

##为MM>0.8，GS>0.5或者0.6

# 指定datTrait中感兴趣的一个性状

traitSelect = as.data.frame(datTraits$ESTIMATEScore_estimate)

names(traitSelect) = "ESTIMATEscore"

# 计算MM的P值

geneModuleMembership = as.data.frame(cor(datExpr, MEs, use = "p"))

MMPvalue = as.data.frame(corPvalueStudent(as.matrix(geneModuleMembership ), nSamples))

names(geneModuleMembership) = paste("MM", modNames, sep="")

names(MMPvalue) = paste("p.MM", modNames, sep="")

# 计算性状和基因表达量之间的相关性（GS）

geneTraitSignificance = as.data.frame(cor(datExpr,traitSelect, use = "p"))

GSPvalue = as.data.frame(corPvalueStudent(as.matrix(geneTraitSignificance),

nSamples))

names(geneTraitSignificance) = paste("GS.", names(traitSelect), sep="")

names(GSPvalue) = paste("p.GS.", names(traitSelect), sep="")

#筛选hub gene

#MEs和MEs0是同一个文件，一个是模块颜色，一个是模块编号，看对应关系

hub<- abs(geneModuleMembership$MMgrey) > 0.8 & abs(geneTraitSignificance) > 0.5

table(hub)

hubgene_grey <- dimnames(data.frame(datExpr))[[2]][hub]

#构建散点图矩阵

##column是感兴趣的色块所在的列 MM<-abs(geneModuleMembership[moduleGenes,column])

MM < -abs(geneModuleMembership[moduleGenes,1])

GS <- abs(geneTraitSignificance[moduleGenes, 1])

c<-as.data.frame(cbind(MM,GS))

rownames(c)=brown_module$genename

head(c)

#对基因进行分组

# hub基因和module内全部基因进行匹配，匹配成功返回1，没有匹配到的返回0

match<- brown_module$genename %in% hubgene_brown

# 将匹配信息添加到散点图矩阵最后一列

c$group<-match

head(c)

#绘制散点图

library(ggplot2)

pdf("MM vs. GS_blue_TL.pdf",width = 7,height = 7)

ggplot(data=c, aes(x=MM, y=GS,color=group))+geom_point(size=1.5)+

scale_colour_manual(values=c("grey60","#DE6757"))+ theme_bw()+

theme(panel.grid.major = element_blank(),panel.grid.minor = element_blank())+

labs(x="Module Membership in blue module", y="Gene significance for TL",

title = "Module membership vs. gene significance ")+

theme(axis.title.x =element_text(size=14), axis.title.y=element_text(size=14),

axis.text = element_text(size = 12),

axis.text.x = element_text(colour = "black"),

axis.text.y = element_text(colour = "black"),

plot.title = element_text(hjust = 0.5,size = 16,face = "bold"),

plot.margin = unit(rep(2,4),'lines')) +

theme(legend.position = 'none')+

geom_hline(aes(yintercept=0.6),colour="#5B9BD5",lwd=1,linetype=5)+

geom_vline(aes(xintercept=0.8),colour="#5B9BD5",lwd=1,linetype=5)

######module基因做单因素cox分析#####

library(survival)

library(survminer)

Survival_model <- datExpr

Survival_model$sample <- rownames(Survival_model)

Survival_model <- merge(LUAD_Survival[which(LUAD_Survival$sample %in% rownames(datExpr)),],

Survival_model,by = "sample")

names(Survival_model) <- gsub("-","_",names(Survival_model))

covariates <- grey_module$genename

covariates <- gsub("\\.","_",covariates)##几个基因名不匹配

model_univ <- sapply(covariates,

function(x) as.formula(paste('Surv(OS.time,OS)~', x)))

model_univ <- lapply(model_univ, function(x) {coxph(x, data = Survival_model)})

model_univ <- lapply(model_univ,

function(x){

x <- summary(x)

#获取p值

p.value<-signif(x$wald["pvalue"], digits=2)

#获取HR

HR <-signif(x$coef[2], digits=2);

#获取95%置信区间

HR.confint.lower <- signif(x$conf.int[,"lower .95"], 2)

HR.confint.upper <- signif(x$conf.int[,"upper .95"],2)

HR <- paste0(HR, " (",

HR.confint.lower, "-", HR.confint.upper, ")")

res<-c(p.value,HR)

names(res)<-c("p.value","HR (95% CI for HR)")

return(res)

})

#转换成数据框，并转置

res_model <- t(as.data.frame(model_univ,check.names = FALSE))

res_model <- as.data.frame(res_model)

res_model

write.csv(res_model,file = "Data/Modelgene Univariate Analysis.csv")

names(res_model)[1] <- "pvalue"

hubGene_univ <- rownames(res_model[which(res_model$pvalue <0.05),])

######lasso分析#####

library(survival)

library(glmnet)

#生存状态和生存时间需要转化为double类型

Survival_model$OS <- as.double(Survival_model$OS)

Survival_model$OS.time <- as.double(Survival_model$OS.time)

rownames(Survival_model) <- Survival_model$sample

dep_var <- data.matrix(Surv(time = Survival_model$OS.time,

event = Survival_model$OS))#设置因变量

Lasso_exp <- colnames(Survival_model) %in% hubGene_univ

marker_exp <- Survival_model[,Lasso_exp]#表达谱

#构建模型

fit_lasso <- glmnet(x = marker_exp,dep_var,family = "cox",alpha = 1)

plot(fit_lasso,xvar = "lambda")

#交叉验证 #源代码无data.matrix会报错

set.seed(16)

lasso_fit <- cv.glmnet(x = data.matrix(marker_exp),dep_var,nfolds = 10,

family ="cox",alignment = "lambda")

plot(lasso_fit)

#筛选变量

coefficient <- coef(lasso_fit,s = lasso_fit$lambda.min)

Active_index <- which(as.numeric(coefficient)!=0)

active_coefficients <- as.numeric(coefficient)[Active_index]

si_gene_multi_cox <- rownames(coefficient)[Active_index]

si_gene_multi_cox

######多因素multivariate-cox#####

#fit_mul <- coxph(Surv(OS.time,OS)~CISD1+DDIT4+DECR1+GLS2+HERPUD1+OTUB1+PEBP1+PIR+PPP1R13L+YWHAE,

# x = T, y = T, data = Survival_model)#原

#fit_mul <- coxph(Surv(OS.time,OS)~CA9+GLS2+PEBP1+PIR+PPP1R13L+DECR1+OTUB1+GDF15+DDIT4,

# x = T, y = T, data = Survival_model)#1se16多因素选p < 0.05加到cindex > 0.7，9个

fit_mul <- coxph(Surv(OS.time,OS)~PIR+PEBP1+PPP1R13L+CA9+GLS2+DECR1+OTUB1+YWHAE,

x = T, y = T, data = Survival_model)#min16，8个

sumfit <- summary(fit_mul)

sumfit$conf.int

sumfit$coefficients

#Determine the C-index

c.index<-t(as.data.frame(sumfit$concordance))

c.index

Low95 <- (c.index[1]) - 1.96*(c.index[2])

Upper95 <-(c.index[1]) + 1.96*(c.index[2])

c.index<-cbind(c.index[1], Low95, Upper95)

c.index

#1se16:CA9+CISD1+DDIT4+DECR1+GCLC+GDF15+GLS2+HSF1+IL33+OTUB1+PEBP1+PIR+PPP1R13L+YWHAE

#min16:ALOX15+ANGPTL7+BCAT2+FTMT+HNF4A+CA9+CISD1+DDIT4+DECR1+GCLC+GDF15+GLS2+HSF1+IL33+OTUB1+PEBP1+PIR+PPP1R13L+YWHAE

#####生存曲线#####

library(rms)

library(survival)

hubgenes <- c("PIR","PEBP1","PPP1R13L","CA9","GLS2","DECR1","OTUB1","YWHAE")

pred.multinom <- predict(fit_mul,

Survival_model[,which(colnames(Survival_model) %in% hubgenes)])

pred.multinom <- as.data.frame(pred.multinom)

pred.multinom$sample <- rownames(pred.multinom)

Survival_group <- merge(dplyr::select(Survival_model,c(1,2,4)),pred.multinom,by="sample")

#要有行名，会带着下面的都出现行名

rownames(Survival_group) <- Survival_group$sample

#取界值

library(survival)

library(survminer)

res.cut <- surv_cutpoint(Survival_group, time = "OS.time", event = "OS",

variables = "pred.multinom")

summary(res.cut)

#按界值分组

res.cat <- surv_categorize(res.cut)

head(res.cat)

table(res.cat$pred.multinom)

#自行分组

Survival_group <- dplyr::arrange(Survival_group,pred.multinom)

Survival_group$group <- "High Score"

Survival_group$group[1:241] <- "Low Score"

#拟合生存曲线

fit_cut <- survfit(Surv(OS.time, OS) ~group, data = Survival_group)

#conf.int表示上下的可信区间

ggsurvplot(fit_cut, data=Survival_group, linetype = 1,

palette = c("#EE0000B2","#3B4992B2"),

size=1,surv.scale = c("percent"),pval = TRUE,legend.title = "",

legend.labs = c("High Scores", "Low Scores"),

break.time.by =12,

xlim = c(0,120),

risk.table = T,risk.table.title = "Patients at risk",

ylab = "Overall Survival, %",

xlab = "Months",font.x = c(20,"plain","black"),

font.y = c(20,"plain","black"),font.tickslab = c(20,"plain","black"),

risk.table.fontsize = 6.5,font.legend =c(25,"plain","black"),

font.main = c(20,"plain","black"),pval.size = 10)

GroupHigh <- rownames(Survival_group)[which(Survival_group$group=="High Score")]

GroupLow <- rownames(Survival_group)[which(Survival_group$group=="Low Score")]

#####GEO生存验证#####

library(survival)

library(survminer)

GEO_Surv <- read.csv("D:/AaPaper/Date整理/GEO/LUAD/LUAD_Ferro/3.csv")#6个队列分别验证

GEO_Surv$X <- NULL

rownames(GEO_Surv) <- GEO_Surv$accession

pred_GEO <- predict(fit_mul,GEO_Surv)

head(pred_GEO)

#表达矩阵要有行名，predict函数行名会用表达矩阵的行名

pred_GEO <- as.data.frame(pred_GEO)

pred_GEO$accession <- rownames(pred_GEO)

GEO_Surv <- merge(GEO_Surv,pred_GEO,by = "accession")

res.cut_GEO <- surv_cutpoint(GEO_Surv, time = "time", event = "status",

variables = "pred_GEO")

summary(res.cut_GEO)

#按界值分组

res.cat_GEO <- surv_categorize(res.cut_GEO)

head(res.cat_GEO)

table(res.cat_GEO$pred_GEO)

#自行分组

GEO_Surv <- dplyr::arrange(GEO_Surv,pred_GEO)

GEO_Surv$group <- "High Score"

GEO_Surv$group[1:29] <- "Low Score"

#拟合生存曲线

fit_GEO <- survfit(Surv(time, status) ~group, data = GEO_Surv)

#conf.int表示上下的可信区间

ggsurvplot(fit_GEO, data=GEO_Surv, linetype = 1,

palette = c("#EE0000B2","#3B4992B2"),

size=1,surv.scale = c("percent"),pval = TRUE,legend.title = "",

legend.labs = c("High Scores", "Low Scores"),

break.time.by =12,

xlim = c(0,120),

risk.table = T,risk.table.title = "Patients at risk",

ylab = "Overall Survival, %",

xlab = "Months",font.x = c(20,"plain","black"),

font.y = c(20,"plain","black"),font.tickslab = c(20,"plain","black"),

risk.table.fontsize = 6.5,font.legend =c(25,"plain","black"),

font.main = c(20,"plain","black"),pval.size = 10)

#####time RoC curve######

library(survival)

library(timeROC)

#GEO数据:GEO_Surv

#TCGA数据:Survival_group

ROC_TCGA <- timeROC(T=Survival_group$OS.time, #事件时间

delta=Survival_group$OS, ##事件状态

marker=Survival_group$pred.multinom,##默认marker 值越大。事件越可能发生，相反的话需要加负号

cause=1, #所关心的事件结局，死在事件是1

weighting="marginal", ##"marginal", "cox","aalen"分别是KM,cox,additive Aalen模型

times=c(12,24,36,48,60), ##1.2.3年ROC

ROC=TRUE) ##保存灵敏度和特异度的值

ROC_TCGA

plot(ROC_TCGA, time=12, col="#D86779", title=FALSE, lwd=2)

plot(ROC_TCGA, time=24, col="#21A2A2",add=TRUE,title=FALSE, lwd=2)

plot(ROC_TCGA, time=36, col="#E3B227", add=TRUE, title=FALSE, lwd=2)

legend(x=0.5, y=0.55,

c(paste0("AUC at 1 years: ", round (ROC_TCGA$AUC[1],4)),

paste0("AUC at 2 years: ", round (ROC_TCGA$AUC[2],4)),

paste0("AUC at 3 years: ", round (ROC_TCGA$AUC[3],4))),

col=c("#D86779","#21A2A2","#E3B227") , lwd=2,bty="n")

#lty:line type。可以是数字或字符c(0="blank",1="solid" (default), 2 ="dashed" , 3 ="dotted",4= ）

#lwd: line width,默认是1

#bty:图例框是否输出，0为画出，默认为n不画出

GEO_Surv <- read.csv("D:/AaPaper/Date整理/GEO/LUAD/LUAD_Ferro/6.csv")#6个队列分别验证

GEO_Surv$X <- NULL

rownames(GEO_Surv) <- GEO_Surv$accession

pred_GEO <- predict(fit_mul,GEO_Surv)

head(pred_GEO)

#表达矩阵要有行名，predict函数行名会用表达矩阵的行名

pred_GEO <- as.data.frame(pred_GEO)

pred_GEO$accession <- rownames(pred_GEO)

GEO_Surv <- merge(GEO_Surv,pred_GEO,by = "accession")

ROC_GEO <- timeROC(T=GEO_Surv$time, #事件时间

delta=GEO_Surv$status, ##事件状态

marker=GEO_Surv$pred_GEO,##默认marker 值越大。事件越可能发生，相反的话需要加负号

cause=1, #所关心的事件结局，死在事件是1

weighting="marginal", ##"marginal", "cox","aalen"分别是KM,cox,additive Aalen模型

times=c(12,24,36,48,60), ##1.2.3年ROC

ROC=TRUE) ##保存灵敏度和特异度的值

#GEO数据:GEO_Surv

#TCGA数据:Survival_cut

ROC_GEO

plot(ROC_GEO, time=12, col="#D86779", title=FALSE, lwd=2)

plot(ROC_GEO, time=24, col="#21A2A2",add=TRUE,title=FALSE, lwd=2)

plot(ROC_GEO, time=36, col="#E3B227", add=TRUE, title=FALSE, lwd=2)

legend(x=0.5, y=0.55,

c(paste0("AUC at 1 years: ", round (ROC_GEO$AUC[1],4)),

paste0("AUC at 2 years: ", round (ROC_GEO$AUC[2],4)),

paste0("AUC at 3 years: ", round (ROC_GEO$AUC[3],4))),

col=c("#D86779","#21A2A2","#E3B227") , lwd=2,bty="n")

#####单基因生存#####

library(survminer)

Survival_singlegene <- dplyr::select(Survival_model,c(2,4,67,98,137,240,247,257,265,360))

Survival_singlegene <- dplyr::arrange(Survival_singlegene,OTUB1)

Survival_singlegene$Group <- "High Expression"

Survival_singlegene$Group[1:241] <- "Low Expression"

fit_singlegene <- survfit(Surv(OS.time, OS) ~ Group, data = Survival_singlegene)

ggsurvplot(fit_singlegene, data=Survival_singlegene, linetype = 1,

palette = c("#EE0000B2","#3B4992B2"),

size=1,surv.scale = c("percent"),pval = TRUE,legend.title = "",

legend.labs = c("High Expression","Low Expression"),

break.time.by =12,

xlim = c(0,120),title="OTUB1",

risk.table = F,risk.table.title = "Patients at risk",

ylab = "Overall Survival, %",

xlab = "Months",font.x = c(20,"plain","black"),

font.y = c(20,"plain","black"),font.tickslab = c(20,"plain","black"),

risk.table.fontsize = 6.5,font.legend =c(25,"plain","black"),

font.main = c(20,"plain","black"),pval.size = 10)

#####计算免疫评分的差异#####

score_high <- Score[which(Score$ID %in% GroupHigh),]

score_low <- Score[which(Score$ID %in% GroupLow),]

score_long <- rbind(score_high,score_low)

score_long <- score_long %>% gather(key = scores,value = value,2:5)

score_long$Group <- "High Score"

score_long$Group[which(score_long$ID %in% GroupLow)] <- "Low Score"

library(ggpubr)

ggplot(data = score_long,aes(x = scores,y = value,fill = Group),

outlier.shape = NA)+

geom_boxplot() + theme_minimal()+

scale_fill_manual(values = c("#EE3A8C","#00B2EE"))+

stat_compare_means(label = "p.format",method = "t.test",hide.ns = T)+

theme(axis.text.x = element_text(size = 12,angle = 0,hjust = 0.5,vjust = 0.5),

axis.title.x = element_text(size = 15),

axis.title.y = element_text(size = 15),legend.position = "right")

#小提琴图

library(ggpubr)

#1/2/3

ggviolin(score_long[1:1446,],x = "scores",y = "value",color="Group",

xlab = "",ylab = "Scores",main="",

palette = c("#B10E0E","#10129E"),add = "boxplot",outlier.shape=NA)+

stat_compare_means(aes(group=Group),label = "p",

method = "t.test", hide.ns = T,

cex=5,hjust=0.5,vjust = -2)+

theme(axis.text.x = element_text(angle = 0,hjust = 0.5,vjust = 1))

#Tumor Purity

ggviolin(score_long[1447:1928,],

x = "Group",y = "value",color="Group",

xlab = "",ylab = "Tumor Purity",main="",

palette = c("#B10E0E","#10129E"),add="boxplot",outlier.shape=NA)+

stat_compare_means(aes(group=Group),label = "p",

method = "t.test", hide.ns = T,

cex=5,hjust=-1,vjust = -1)+

theme(axis.text.x = element_text(angle = 0,hjust = 0.5,vjust = 1))

#Stromal Score

ggviolin(score_long[1:482,],

x = "Group",y = "value",color="Group",

xlab = "",ylab = "Stromal Score",main="",

palette = c("#B10E0E","#10129E"),add="boxplot",outlier.shape=NA)+

stat_compare_means(aes(group=Group),label = "p.signif",

method = "t.test", hide.ns = T,

cex=10,hjust=-2.5,vjust = 0)+

theme(axis.text.x = element_text(angle = 0,hjust = 0.5,vjust = 1))

#Immune Score

ggviolin(score_long[483:964,],

x = "Group",y = "value",color="Group",

xlab = "",ylab = "Immune Score",main="",

palette = c("#B10E0E","#10129E"),add="boxplot",outlier.shape=NA)+

stat_compare_means(aes(group=Group),label = "p.signif",

method = "t.test", hide.ns = T,

cex=10,hjust=-2.5,vjust = 0)+

theme(axis.text.x = element_text(angle = 0,hjust = 0.5,vjust = 1))

#Estimate Score

ggviolin(score_long[965:1446,],

x = "Group",y = "value",color="Group",

xlab = "",ylab = "Estimate Score",main="",

palette = c("#B10E0E","#10129E"),add="boxplot",outlier.shape=NA)+

stat_compare_means(aes(group=Group),label = "p.signif",

method = "t.test", hide.ns = T,

cex=10,hjust=-2.5,vjust = 0)+

theme(axis.text.x = element_text(angle = 0,hjust = 0.5,vjust = 1))

#####差异基因+lncRNA#####

library(edgeR)

#筛选编码蛋白基因

GeneType <- read.csv("D:/AaPaper/Date整理/GeneType.csv")

Protein_Coding <- GeneType[which(GeneType$gene_type =="protein_coding"),]

lncName <- GeneType[which(GeneType$gene_type == "lincRNA"),]

#去掉名字版本细节

lncName$gene_name <- gsub("\\..*$","",lncName$gene_name)

#分组

#样本需要按分组排序

#Exp_High <- LUAD_TPM[,which(colnames(LUAD_TPM) %in% GroupHigh)]

#Exp_Low <- LUAD_TPM[,which(colnames(LUAD_TPM) %in% GroupLow)]

#LUAD_DEGExp <- cbind(Exp_High,Exp_Low)

#edgR差异基因

LUAD_Counts <- read.csv("D:/AaPaper/Date整理/TCGA/LUAD/LUAD_Counts.csv")

LUAD_Counts <- LUAD_Counts %>% column_to_rownames("X")

#排序

Counts_High <- LUAD_Counts[,which(colnames(LUAD_Counts) %in% GroupHigh)]

Counts_Low <- LUAD_Counts[,which(colnames(LUAD_Counts) %in% GroupLow)]

LUAD_Counts <- cbind(Counts_High,Counts_Low)

#edgR

group <- c(rep("1",241),rep("0",241))

design <- model.matrix(~group)

LUAD_Counts <- 2^LUAD_Counts-1 #还原数据

LUAD_Counts <- as.matrix(LUAD_Counts)

Counts_pro <- LUAD_Counts[which(rownames(LUAD_Counts) %in% Protein_Coding$gene_name),]

y <- DGEList(counts = Counts_pro,group = group)

y <- calcNormFactors(y)#标准化数据

y <- estimateCommonDisp(y)#普通离散度

y <- estimateTagwiseDisp(y)#基因间范围内的离散度

et <- exactTest(y,pair = c("1","0"))#精确检验

ordered_tags <- topTags(et,n = 100000)

deg <- as.data.frame(ordered_tags)

deg$Group <- "not-significant"

deg$Group[which((deg$PValue < 0.05) & (deg$logFC > 1))] <- "Upregulated"

deg$Group[which((deg$PValue < 0.05) & (deg$logFC < -1))] <- "Downregulated"

deg$logP<- -log10(deg$PValue)

table(deg$Group)#protein-coding mrna

#deg <- dplyr::arrange(deg,-logFC)#添加负号升序

write.csv(deg[deg$Group!="not-significant",],file = "Plot/PPI/deg.csv")

#火山图

library(ggpubr)

deg$ID <- rownames(deg)

deg <- deg[order(deg$PValue),]

up.genes <- head(deg$ID[which(deg$Group =="Upregulated")],10)

down.genes <- head(deg$ID[which(deg$Group =="Downregulated")],10)

deg.top10.genes <- c(as.character(up.genes),as.character(down.genes))

deg$Label = ""

deg$Label[match(deg.top10.genes,deg$ID)]<-deg.top10.genes

ggscatter(deg,x="logFC",y="logP",

color = "Group",

palette = c("#1f1fc8","gray","#D83131"),

label = deg$Label,

font.label = 10,

repel = T,

size = 2,

alpha = 0.7,

ylab = "-log10(P-Value)")+

geom_hline(yintercept = 1.3010,linetype = "dashed") +

geom_vline(xintercept = c(-1,1),linetype = "dashed")+

theme_minimal()+

theme(axis.text.x = element_text(size = 15, angle = 0, hjust = 0.5, vjust = 3.0),

axis.text.y = element_text(size = 15),

axis.title.x = element_text(size = 15),

axis.title.y = element_text(size = 15),legend.position = "right")

#lncRNA

Counts_lnc <- LUAD_Counts[which(rownames(LUAD_Counts) %in% lncName$gene_name),]

#group <- c(rep("1",211),rep("0",271))

y2 <- DGEList(counts = Counts_lnc,group = group)

y2 <- calcNormFactors(y2)#标准化数据

y2 <- estimateCommonDisp(y2)#普通离散度

y2 <- estimateTagwiseDisp(y2)#基因间范围内的离散度

et2 <- exactTest(y2,pair = c("1","0"))#精确检验

ordered_tags2 <- topTags(et2,n = 100000)

delncRNA <- as.data.frame(ordered_tags2)

delncRNA$Group = "not-significant"

delncRNA$Group[which((delncRNA$PValue<0.05) & (delncRNA$logFC > 1))] = "Upregulated" #up in HS group

delncRNA$Group[which((delncRNA$PValue<0.05) & (delncRNA$logFC < -1))] = "Downregulated"

delncRNA$logP<- -log10(delncRNA$PValue)

table(delncRNA$Group)

write.csv(delncRNA[delncRNA$Group!="not-significant",],file = "Data/DElncRNA.csv")

#火山图

library(ggpubr)

delncRNA$ID <- rownames(delncRNA)

delncRNA <- delncRNA[order(delncRNA$PValue),]

up.lncRNA <- head(delncRNA$ID[which(delncRNA$Group =="Upregulated")],10)

down.lncRNA <- head(delncRNA$ID[which(delncRNA$Group =="Downregulated")],10)

deg.top10.lncRNA <- c(as.character(up.lncRNA),as.character(down.lncRNA))

delncRNA$Label = ""

delncRNA$Label[match(deg.top10.lncRNA,delncRNA$ID)]<-deg.top10.lncRNA

ggscatter(delncRNA,x="logFC",y="logP",

color = "Group",

palette = c("#1f1fc8","gray","#D83131"),

label = delncRNA$Label,

font.label = 10,

repel = T,

size = 2,

alpha = 0.7,

ylab = "-log10(P-Value)")+

geom_hline(yintercept = 1.3010,linetype = "dashed") +

geom_vline(xintercept = c(-1,1),linetype = "dashed")+

theme_minimal()+

theme(axis.text.x = element_text(size = 15, angle = 0, hjust = 0.5, vjust = 3.0),

axis.text.y = element_text(size = 15),

axis.title.x = element_text(size = 15),

axis.title.y = element_text(size = 15),legend.position = "right")

#####miRNA#####格式是log TPM，只能limma

library(limma)

miRNA <- read.csv("D:/AaPaper/Date整理/TCGA/LUAD/LUAD_miRNA.csv")

rownames(miRNA) <- miRNA$X

miRNA <- miRNA[,which(colnames(miRNA) %in% rownames(datExpr))]

#排序

miRNA_High <- miRNA[,which(colnames(miRNA) %in% GroupHigh)]

miRNA_Low <- miRNA[,which(colnames(miRNA) %in% GroupLow)]

miRNA <- cbind(miRNA_High,miRNA_Low)

#分组

Group_DEmiRNA <- data.frame("HS" = c(rep(1,208),rep(0,208)),"LS" = c(rep(0,208),rep(1,208)))

rownames(Group_DEmiRNA)<-c(colnames(miRNA_High),colnames(miRNA_Low))

#差异分析

fit_DEmiRNA <- lmFit(miRNA,Group_DEmiRNA,method = "ls")#线性拟合

contrast.matrix <- makeContrasts(HS - LS,levels=Group_DEmiRNA)#确定比较两组

fit_DEmiRNA <- contrasts.fit(fit_DEmiRNA,contrast.matrix)

fit_DEmiRNA <- eBayes(fit_DEmiRNA)#经验贝叶斯公式计算拟合标准误差

#p值矫正，提取差异分析结果，Toptable

all_diff_miRNA <- topTable(fit_DEmiRNA, adjust.method = 'fdr',coef=1,p.value = 1,lfc <- log(1,2),number = 60000,sort.by = 'logFC')

all_diff_miRNA$logP<- -log10(all_diff_miRNA$P.Value)

all_diff_miRNA$ID<-rownames(all_diff_miRNA)

all_diff_miRNA$Group = "not-significant"

#将P.Val<0.05,logFC>0.5的基因设置为显著上调基因

#将P.Val<0.05,logFC<0.5的基因设置为显著下调基因

all_diff_miRNA$Group[which((all_diff_miRNA$P.Value < 0.05) & (all_diff_miRNA$logFC > 0.5))] = "Upregulated in HS group"

all_diff_miRNA$Group[which((all_diff_miRNA$P.Value < 0.05) & (all_diff_miRNA$logFC < -0.5))] = "Downregulated in HS group"

table(all_diff_miRNA$Group)

library(ggpubr)

#all_diff_miRNA$ID <- rownames(all_diff_miRNA)

all_diff_miRNA <- all_diff_miRNA[order(all_diff_miRNA$P.Value),]

up.miRNA <- head(all_diff_miRNA$ID[which(all_diff_miRNA$Group =="Upregulated in HS group")],10)

down.miRNA <- head(all_diff_miRNA$ID[which(all_diff_miRNA$Group =="Downregulated in HS group")],10)

deg.top10.miRNA <- c(as.character(up.miRNA),as.character(down.miRNA))

all_diff_miRNA$Label = ""

all_diff_miRNA$Label[match(deg.top10.miRNA,all_diff_miRNA$ID)]<-deg.top10.miRNA

ggscatter(all_diff_miRNA,x="logFC",y="logP",

color = "Group",

palette = c("#1f1fc8","gray","#D83131"),

label = all_diff_miRNA$Label,

font.label = 10,

repel = T,

size = 2,

alpha = 0.7,

ylab = "-log10(P-Value)")+

geom_hline(yintercept = 1.3010,linetype = "dashed") +

geom_vline(xintercept = c(-0.5,0.5),linetype = "dashed")+

theme_minimal()+

theme(axis.text.x = element_text(size = 15, angle = 0, hjust = 0.5, vjust = 3.0),

axis.text.y = element_text(size = 15),

axis.title.x = element_text(size = 15),

axis.title.y = element_text(size = 15),legend.position = "right")

#####ceRNA network#####

NameDEmiRNAup <- all_diff_miRNA$ID[all_diff_miRNA$Group == "Upregulated in HS group"]

NameDEmiRNAup <- paste0("hsa-",NameDEmiRNAup)

NameDEmiRNAdown <- all_diff_miRNA$ID[all_diff_miRNA$Group == "Downregulated in HS group"]

NameDEmiRNAdown <- paste0("hsa-",NameDEmiRNAdown)

NameDEproteindown <- deg$ID[deg$Group=="Downregulated"]

NameDEproteinup <- deg$ID[deg$Group=="Upregulated"]

NameDElncdown <- delncRNA$ID[delncRNA$Group=="Downregulated"]

NameDElncup <- delncRNA$ID[delncRNA$Group=="Upregulated"]

##miRcode lncRNA

miRcode <- read.table("D:/AaPaper/LUAD/数据/mircode_highconsfamilies.txt",

sep = "\t",header = T,quote = "",fill = T,comment.char = "!",

stringsAsFactors = FALSE)

miRcode <- dplyr::select(miRcode,1,2,3,4)

#table(miRcode$gene_class)

miRcode_uplnc <- miRcode[which(miRcode$gene_symbol %in% NameDElncup),]

miRcode_downlnc <- miRcode[which(miRcode$gene_symbol %in% NameDElncdown),]

##multiMiR

library(multiMiR)

#联网检索，跟网速有关

mRNA_miRNA <- get_multimir(org = "hsa",target = NameDEprotein,

mirna = NameDEmiRNA,

table = "mirtarbase",#搜索的数据库，单个数据库或"validated","predicted","disease.drug","all"

summary = T)

mRNA_miRNAcorr <- mRNA_miRNA@data

mRNA_miRNAsum <- mRNA_miRNA@summary

#apply(mRNA_miRNA@summary[,6:10],2,sum)

mirTarbase_upmiRNA <- mRNA_miRNAcorr[mRNA_miRNAcorr$mature_mirna_id %in% NameDEmiRNAup,]

mirTarbase_downmiRNA <- mRNA_miRNAcorr[mRNA_miRNAcorr$mature_mirna_id %in% NameDEmiRNAdown,]

######功能富集#####

###1.GO

# GO三大注释——BP:生物学过程 CC:细胞学组分 MF：分子生物学功能

#ENTREZID: 4312 8318

#SYMBOL: MMP1 CDC45

#ENSEMBL: ENSG00000196611 ENSG00000093009

library(clusterProfiler)

library(org.Hs.eg.db)

DEGname <- as.character(deg[deg$Group != "not-significant",]$ID)

#基因ID类型为ENSEMBL的ID形式，选择BP功能组(BP/CC/MF)

#c("PI3","LCE2A","SPRR2E","SPRR2A","SPRR2G","SPRR2B","LCE3D","LCE3E","RPTN")

Go_resultBP <- enrichGO(DEGname,'org.Hs.eg.db',

keyType = "SYMBOL",

ont="BP",

pvalueCutoff=0.05)

Go_resultCC <- enrichGO(DEGname, 'org.Hs.eg.db',

keyType = "SYMBOL",

ont="CC",

pvalueCutoff=0.05)

Go_resultMF <- enrichGO(DEGname, 'org.Hs.eg.db',

keyType = "SYMBOL",

ont="MF",

pvalueCutoff=0.05)

#enrichplot::cnetplot(Go_resultBP,showCategory = 10,circular = T,colorEdge = T) #B站看到的代码

barplot(Go_resultBP, showCategory=10)

barplot(Go_resultCC, showCategory=10)

barplot(Go_resultMF, showCategory=10)

dotplot(Go_resultBP, showCategory=10)

###2.KEGG(需要用ENTREZID形式的数据)

library(org.Hs.eg.db)

library(clusterProfiler)

#将基因名转换为ENTREZID格式

DEGname <- bitr(DEGname, fromType="SYMBOL", toType=c("ENTREZID"), OrgDb="org.Hs.eg.db")

DEGname <- DEGname$ENTREZID

enrich_KEGG <- enrichKEGG(DEGname,

organism = "hsa",

pvalueCutoff = 0.05)

barplot(enrich_KEGG, showCategory=10)

dotplot(enrich_KEGG, showCategory=10)

#####免疫细胞ssGSEA#####

library(GSVA)

ImmuneGeneSet <- read.csv("D:/AaPaper/Date整理/geneset(immune).csv")

#gsvasig<-lapply(gsvasig, function(x) x[!is.na(x)])

#基因表达一个行是基因，列是样本的matrix

Immunecellresult <- GSVA::gsva(as.matrix(LUADFerro),ImmuneGeneSet,

method = "ssgsea",min.sz = 1,max.sz = Inf,

mx.diff=TRUE,verbose=FALSE, parallel.sz=0)

Immunecellresult <- as.data.frame(t(Immunecellresult))

#GSVA_long <- cbind(rownames(GSVAresult),GSVAresult[,c(2,3,4,7,13,15,16,23,25,26)])

GSVA_long <- cbind(rownames(Immunecellresult),Immunecellresult)

names(GSVA_long)[1] <- "sample"

GSVA_long$Group <- "High"

GSVA_long$Group[which(GSVA_long$sample %in% GroupLow)] <- "Low"

GSVA_long <- tidyr::gather(GSVA_long,key = Cell_type,value = Proportion,2:25)

library(ggpubr)

ggpubr::ggboxplot(GSVA_long,outlier.shape = NA,

x = "Cell_type",y = "Proportion",

color = "black",fill = "Group",

group = "Group",size = 0.3,palette = c("#E741AD","#1CAAC6"),

xlab = "",ylab = "Enrichment Score") +

stat_compare_means(aes(group=Group),label = "p.signif",

method = "t.test",hide.ns = T,cex=5)+

theme(axis.text.x = element_text(angle = 60,hjust = 1,vjust = 1,size = 7.5))

#相互关系

library(ggplot2)

library(ggcorrplot)

library(ggthemes)

Immune_corr<-as.data.frame(round(cor(Immunecellresult,method = "spearman"),3))##默认pearson

Immune_p.mat<-as.data.frame(cor_pmat(Immunecellresult))

ggcorrplot(Immune_corr,hc.order=TRUE,hc.method="complete",

outline.col='white',ggtheme = theme_bw(),type = "upper",

lab=TRUE,lab_size=1.5,p.mat=Immune_p.mat,insig="blank",tl.cex = 7.5)

#tl.cex坐标轴文本大小

#####免疫检查点#####

ImmuneCP <- c("IDO1","CTLA4","TNFRSF9","ICOS","CD80","TIGIT","CD70","TNFSF9",

"CD86","POCD1","LAIR1","TNFRSF8","TNFSF15","TNFRSF14","CD276","CD40",

"TNFRSF4","TNFSF14","HLA2","CD244","CD274","HAVCR2","CD27","BTLA",

"LGALS9","CD28","CD48","TNFRSF25","CD40LG","VTCN1","CD160","CD44",

"TNFSF18","TNFRSF18","BTNL2","CD200R1","TNFSF4","CD200","NRP1")

CP_exp <- LUADFerro[rownames(LUADFerro) %in% ImmuneCP,]

CP_exp <- as.data.frame(t(CP_exp))

CP_exp <- cbind(rownames(CP_exp),CP_exp)

names(CP_exp)[1] <- "sample"

CP_exp$Group <- "High Score"

CP_exp$Group[which(CP_exp$sample %in% GroupLow)] <- "Low Score"

CP_exp <- tidyr::gather(CP_exp,key = Gene_type,value = Expression,2:38)

ggpubr::ggboxplot(CP_exp,outlier.shape = NA,

x = "Gene_type",y = "Expression",

color = "black",fill = "Group",

group = "Group",size = 0.3,palette = c("#E741AD","#1CAAC6"),

xlab = "",ylab = "Gene Expression") +

stat_compare_means(aes(group=Group),label = "p.signif",

method = "t.test",hide.ns = T,cex=4)+

theme(axis.text.x = element_text(angle = 70,hjust = 1,vjust = 1,size = 12))

#####KEGG######

library(GSEABase)

KEGGSet <- getGmt("D:/AaPaper/Date整理/c2.KEGG.gmt")

#GSVA分析

library(GSVA)

kegg <- gsva(expr=as.matrix(LUADFerro), KEGGSet,

method = "ssgsea",min.sz = 1,max.sz = Inf,

mx.diff=TRUE,verbose=FALSE, parallel.sz=0)

#分组

GroupKEGG <- ifelse(colnames(kegg) %in% GroupHigh, "High", "Low")

GroupKEGG <- factor(GroupKEGG,levels = c("High","Low"))

#差异分析

Design_KEGG <- model.matrix(~GroupKEGG)

colnames(Design_KEGG) <- levels(GroupKEGG)

fit_KEGG <- lmFit(kegg,Design_KEGG)

fit_KEGG <- eBayes(fit_KEGG)

KEGGDiff <- topTable(fit_KEGG,adjust='fdr',coef=2,number=Inf)

KEGGDiff <- KEGGDiff[KEGGDiff$adj.P.Val < 0.05,]

KEGGDiffdown <- KEGGDiff[KEGGDiff$logFC > 0,]

KEGGDiffup <- KEGGDiff[KEGGDiff$logFC < 0,]

library(pheatmap)

pheatmap(kegg[rownames(kegg) %in% rownames(KEGGDiffup),],

cluster_cols = F,cluster_rows = T,

show_colnames = F,show_rownames = T,

annotation_col = dplyr::select(Survival_group,5),scale = "row",

treeheight_row = 30,

color=c(colorRampPalette(colors=c("blue","white"))(length(bk)/2)

,colorRampPalette(color=c("white","red"))(length(bk)/2)),

legend_breaks = seq(-1,1,1),breaks = bk)

#####Hallmark gene set#####

library(GSVA)

HallmarkGeneSet <- read.csv("D:/AaPaper/Date整理/Hallmark geneset.csv")

#gsvasig<-lapply(gsvasig, function(x) x[!is.na(x)])

#基因表达一个行是基因，列是样本的matrix

GSVAresult <- GSVA::gsva(as.matrix(LUADFerro),HallmarkGeneSet,

method = "ssgsea",min.sz = 1,max.sz = Inf,

mx.diff=TRUE,verbose=FALSE, parallel.sz=0)

GSVAresult <- as.data.frame(t(GSVAresult))

hall_p <- as.data.frame(matrix(nrow = 50,ncol = 2))

names(hall_p) <- c("geneset","pvalue")

for (i in 1:50){

hallHigh <- hall_heat[i,which(colnames(hall_heat)%in% GroupHigh)]

hallLow <- hall_heat[i,which(colnames(hall_heat)%in% GroupLow)]

ttest <- t.test(hallHigh,hallLow)

p=round(ttest$p.value,3)

hall_p$geneset[i] <- rownames(hall_heat)[i]

hall_p$pvalue[i] <- ttest$p.value

}

hall_pname <- hall_p$geneset[hall_p$pvalue < 0.05]

hall_heat <- as.data.frame(t(GSVAresult))

hall_heat <- hall_heat[rownames(hall_heat) %in% hall_pname,]

rownames(hall_heat) <- gsub("HALLMARK_","",rownames(hall_heat))

bk <- c(seq(-1,0,by=0.01),seq(0.01,1,by=0.01)) #色度条调节

library(pheatmap)

pheatmap(hall_heat,cluster_cols = F,cluster_rows = T,

show_colnames = F,show_rownames = T,

annotation_col = dplyr::select(Survival_group,5),scale = "row",

treeheight_row = 30,

color=c(colorRampPalette(colors=c("blue","white"))(length(bk)/2)

,colorRampPalette(color=c("white","red"))(length(bk)/2)),

legend_breaks = seq(-1,1,1),breaks = bk)

#GSVA_long <- cbind(rownames(GSVAresult),GSVAresult[,c(2,3,4,7,13,15,16,23,25,26)])

hall_long <- cbind(rownames(GSVAresult),GSVAresult)

names(hall_long)[1] <- "sample"

hall_long$Group <- "High Score"

hall_long$Group[which(hall_long$sample %in% GroupLow)] <- "Low Score"

hall_long <- tidyr::gather(hall_long,key = Gene_Set,value = Proportion,2:51)

hall_long$Gene_Set <- gsub("HALLMARK_","",hall_long$Gene_Set)

hall_long <- hall_long[which(hall_long$Gene_Set %in% hall_pname),]

library(ggpubr)

ggboxplot(hall_long,outlier.shape = NA,

x = "Gene_Set",y = "Proportion",

color = "black",fill = "Group",

group = "Group",size = 0.3,palette = c("#E741AD","#1CAAC6"),

xlab = "",ylab = "Enrichment Score") +

stat_compare_means(aes(group=Group),label = "p.signif",

method = "t.test",hide.ns = T,cex=5)+

theme(axis.text.x = element_text(angle = 30,hjust = 1,vjust = 1,size = 10))

######GSEA######

deg_GSEA <- deg[which(deg$Group!="not-significant"),]

deg_GSEA <- dplyr::arrange(deg_GSEA,logFC)#排序

deg_GSEA <- dplyr::select(deg_GSEA,1,7)#logFC和ID列

library(clusterProfiler)

zz <- bitr(deg_GSEA$ID,fromType = "SYMBOL",toType = "ENTREZID",OrgDb = "org.Hs.eg.db")

names(deg_GSEA)[2]<-"SYMBOL"

zz <- merge(deg_GSEA,zz,by="SYMBOL",all=F)

zz <- zz[order(zz$logFC,decreasing = T),] #必须是从大到小

#用于分析的z1为向量，，内容是logFC,名字是etrezid,从大到小排序

z1 <- zz$logFC

names(z1)<-zz$ENTREZID

z2 <- gseKEGG(z1,organism = "hsa")

z3 <- z2[order(z2$enrichmentScore,decreasing=T),]

library(enrichplot)

dotplot(z2,showCategory=20)

gseaplot2(z2,row.names(z3)[1],subplots = 1:2,#显示画几部分

base_size = 20,pvalue_table = F,color = "orange",ES_geom = "line")

#####免疫浸润#####

library(IOBR)

TPM_data <- LUAD_TPM[,which(names(LUAD_TPM) %in% rownames(datExpr))]

ImmuneXcell <- deconvo_tme(eset = 2^TPM_data-1,method = "xcell",arrays = F)#数据必须non-log scale

ImmuneMCP <- deconvo_tme(eset = 2^TPM_data-1,method = "mcpcounter")#数据必须non-log scale

ImmuneCibersort <- deconvo_tme(eset = 2^TPM_data-1,method = "cibersort",

arrays = F,perm = 200)#数据必须non-log scale

ImmuneEpic <- deconvo_tme(eset = 2^TPM_data-1,method = "epic",tumor = T)#数据必须non-log scale

Immune_Analyse <- dplyr::select(Survival_group,1:3)

names(Immune_Analyse)[1] <- "ID"

Immune_Analyse <- merge(Immune_Analyse,ImmuneMCP[,1:11],by="ID")

#names(Immune_Analyse)[4:13] <- gsub("_MCPcounter","",names(Immune_Analyse)[4:13])

#Immune Gene Set

Immune_cell <- cbind(rownames(Immunecellresult),Immunecellresult)

names(Immune_cell)[1] <- "ID"

Immune_Analyse <- merge(Immune_Analyse,Immune_cell,by="ID")

library(survival)

immuneCOX<-function(x){

FML <- as.formula(paste0("Surv(OS.time,OS)~",x))

cox <- coxph(FML, data=Immune_Analyse)

sum <- summary(cox)

sum$coefficients

HR <- round(sum$coefficients[,2],2)

pvalue <- round((sum$coefficients[,5]),3)

CI <- paste0(round(sum$coefficients[,2],2),"(",paste0(round(sum$conf.int[,3:4],2),collapse = "-"),")")

upper <- round(sum$conf.int[,4],2)

lower <- round(sum$conf.int[,3],2)

unicox <- data.frame("characteristics"=x,

"Hazard Ratio"=HR,

"CI95"=CI,

"pvalue"=pvalue,

"upper"=upper,

"lower"=lower)

unicox$Variance<-rownames(unicox)

return(unicox)

}

immuneCOX("Bcells_EPIC")

library(plyr)

#names(Immune_Analyse) <- gsub("\\+","",names(Immune_Analyse)) #xcell

#names(Immune_Analyse) <- gsub("\\(","",names(Immune_Analyse)) #cibersort

Immune_var <- lapply(names(Immune_Analyse)[4:27],immuneCOX)

Immune_var <- ldply(Immune_var,data.frame)

#Immune_var <- Immune_var[c(4,10,15,20,29,34),]

Immune_var<- Immune_var[-7,]

#森林图

Immune_label <- select(Immune_var,c(1,4,3))

#Immune_label$characteristics <- gsub("_EPIC","",Immune_label$characteristics)

#Immune_label$characteristics <- gsub("_xCell","",Immune_label$characteristics)

library(forestplot)

forestplot(Immune_label,#文本部分的数据

mean=Immune_var$Hazard.Ratio,#指定HR

lower=Immune_var$lower, #指定下区间

upper=Immune_var$upper, #指定上区间

zero = 1, #参照竖线取值，一般取在1

lwd.zero=2, ##参照线粗细

lwd.ci=3, ##可信区间线的粗细

lwd.xaxis=2, ##x轴的粗细

boxsize=0.2, ##线中间的方块大小

graph.pos = 3, #设置森林图出现在在表格中的位置

xlab = "", #设置x轴的字

clip=c(0,2),xticks=seq(0,2,by=0.5), #可信区间控制在箭头范围内

lineheight = "auto", ##自动行距

txt_gp = fpTxtGp(ticks = gpar(fontsize =25),xlab = gpar(fontsize =10),label = gpar(fontsize =15)),

col=fpColors(box="#FA7F6F",zero = "#E7DAD2",line="#FFBE7A"))

#森林图（不好看）

library(finalfit)

Immune_fit <- finalfit(Immune_Analyse,names(Immune_Analyse)[3],names(Immune_Analyse)[4:10])

ff_plot(Immune_Analyse,names(Immune_Analyse)[3],names(Immune_Analyse)[4:10])

#####TIDEscore####

TIDE_expression <- LUADFerro

TIDE_expression$mean <- apply(TIDE_expression,1,mean)

TIDE_expression <- TIDE_expression-TIDE_expression$mean

write.csv(TIDE_expression,file = "TIDEexpression.csv")

TIDE_score <- read.csv("Data/TIDEscore.csv")

TIDE_score <- dplyr::select(TIDE_score,c(1,4))

TIDE_score$Group <- "High Score"

TIDE_score$Group[TIDE_score$Patient %in% GroupLow] <- "Low Score"

library(ggpubr)

ggviolin(TIDE_score,x = "Group",y = "TIDE",

color = "black",draw_quantiles = T,ylab = "TIDE Score",xlab = "",

alpha = 0.5,palette = "npg",size = 0.5,fill = "Group",

add = c("boxplot")) +theme_classic2()+

stat_compare_means(method = "t.test",label = "p.signif",

hide.ns = T,size=6.5,vjust = 0.5,

comparisons = combn(unique(TIDE_score$Group),2,simplify = F))+

scale_x_discrete(limits = c("High Score","Low Score"))+

theme(text = element_text(size = 20))

#####Cibersort#####

library(ggplot2)

library(ggcorrplot)

library(ggthemes)

Cibersort_corr<-as.data.frame(round(cor(ImmuneCibersort[,c(2:5,7:23)]),3),method="pearson") ##默认pearson

Cibersort_p.mat<-as.data.frame(cor_pmat(ImmuneCibersort[,c(2:5,7:23)]))

ggcorrplot(Cibersort_corr,hc.order=TRUE,hc.method="complete",

outline.col='white',ggtheme = theme_bw(),type = "upper",

lab=TRUE,lab_size=1.5,p.mat=Cibersort_p.mat,insig="blank",tl.cex = 7.5)

#tl.cex坐标轴文本大小

#####主成分分析#####

model_exp <- Survival_model[,which(colnames(Survival_model) %in% hubgenes)]

write.csv(t(model_exp),file = "Data/modelexp.csv")

#####基因突变######

#####maf文件里核心文件是data,其他是衍生的

library(maftools)

LUADmaf = read.maf(maf = "D:/AaPaper/Date整理/TCGA/LUAD/TCGA-LUAD.maf")

getSampleSummary(LUADmaf)

getGeneSummary(LUADmaf)

datas<-LUADmaf@data

datas<-as.data.frame(datas)

datas$Tumor_Sample_Barcode<-gsub('-','_',datas$Tumor_Sample_Barcode)

##正则表达式去除多余的ID部分

datas$Tumor_Sample_Barcode<-gsub('............$','',datas$Tumor_Sample_Barcode)

##先把data拿出来修改再放回去

data_high<-datas[datas$Tumor_Sample_Barcode %in% GroupHigh,]

data_low<-datas[datas$Tumor_Sample_Barcode %in% GroupLow,]

data_high_maf<-read.maf(data_high)

data_low_maf<-read.maf(data_low)

#####比较两组间差异

mutation_compare <- mafCompare(m1 = data_high_maf, m2 = data_low_maf,

minMut = 5,#只纳入最少n个样本发生突变的基因

m1Name = 'High Score', m2Name = 'Low Score')

print(mutation_compare)

LUAD_diff_mut<-mutation_compare$results[mutation_compare$results$pval<0.05,]

mute_gene <- head(LUAD_diff_mut$Hugo_Symbol,20)

#展示重点变量的总结信息

#Shows sample summry.

getSampleSummary(data_high_maf)

#Plot summarizision

plotmafSummary(maf = data_high_maf, rmOutlier = TRUE, addStat = 'median',

dashboard = TRUE, titvRaw = FALSE)

plotmafSummary(maf = data_low_maf, rmOutlier = TRUE, addStat = 'median',

dashboard = TRUE, titvRaw = FALSE)

#Summarize Transition and Transversions，转换和颠换的占比，SNP

data_low_maf_titv = titv(maf = data_low_maf, plot = FALSE, useSyn = TRUE)

data_high_maf_titv = titv(maf = data_high_maf, plot = FALSE, useSyn = TRUE)

#plot titv summary

plotTiTv(res = data_low_maf_titv)

plotTiTv(res = data_high_maf_titv)

#Lollipop plots，需要有Protein_change这一列，即AACol

#每个图对应一个特定的基因的氨基酸改变情况

lollipopPlot(maf = LUADmaf, gene = 'TP53', AACol = 'HGVSp_Short', showMutationRate = TRUE)

#瀑布图

RColorBrewer::display.brewer.all() ##查看调色板

vc_cols = RColorBrewer::brewer.pal(n = 8, name = 'Paired')

names(vc_cols) = c('Frame_Shift_Del','Missense_Mutation',

'Nonsense_Mutation','Multi_Hit','Frame_Shift_Ins','In_Frame_Ins',

'Splice_Site','In_Frame_Del')

oncoplot(maf=data_high_maf,#top=20,

genes = mute_gene,keepGeneOrder = F,

#top=20可以换成gene=想要的基因，KeepGeneOrder=T默认为False

fontSize = 0.8,#colors = vc_cols,

showTumorSampleBarcodes = F,draw_titv=F,legend_height =6,anno_height =5,

titleFontSize = 1.6,legendFontSize =2,barcode_mar = 0.1,gene_mar = 10)

oncoplot(maf=data_low_maf,#top = 20,

genes = mute_gene,keepGeneOrder = F,

fontSize = 0.8,#colors = vc_cols,

showTumorSampleBarcodes = F,draw_titv=F,legend_height =6 ,anno_height =5,

titleFontSize = 1.6,legendFontSize =2,barcode_mar = 0.1,gene_mar = 10)

#######风险因子累积图#####

#Risk factor风险因子关联图

library(ggplotify)

library(ggplot2)

#biomarker_data整合了生存信息+Score+Group

biomarker_data <- Survival_group

biomarker_data <- biomarker_data %>% remove_rownames() %>%column_to_rownames("sample")

names(biomarker_data)[3] <- "Score"

#biomarker_risk <- biomarker_data

#1.riskscore高低风险分组显示点图

biomarker_data<-biomarker_data[order(as.numeric(biomarker_data$Score)),]

table(biomarker_data$group)

p1=ggplot(data=biomarker_data,aes(x=seq(0,481),y=Score,color=group))+

geom_point()+

scale_x_continuous(breaks=seq(0,481,100))+#x轴范围及间隔,样本数，??必须减1

scale_y_continuous(breaks=seq(-2,2.5,1))+#y轴范围及间隔，评分

geom_hline(aes(yintercept=-0.0325),colour="#BB0000",linetype="dashed")+#y轴标线，评分界值

geom_vline(aes(xintercept=241),colour="#BB0000",linetype="dashed")+#x轴标线

theme(panel.grid.major = element_blank(),

panel.grid.minor = element_blank(),

panel.background = element_blank(),

axis.line=element_line(colour = "black"))+

labs(x="",y="Score")+

theme(axis.line = element_line(size=1, colour = "black"),

axis.text.x = element_text(size = 20,hjust = 0.5,vjust = 0.5),

axis.text.y = element_text(size = 20,hjust = 0.5,vjust = 0.5),

axis.title.x = element_text(size = 23),

axis.title.y = element_text(size = 23),legend.position = "right")

p1

#2.生存状态散点图

biomarker_data$OS=factor(biomarker_data$OS,levels=c("0","1"),labels=c("Alive","Dead"))

#分组排序一下？

p2=ggplot(data=biomarker_data)+

geom_point(aes(x=seq(0,481),y=OS.time,color= OS)) +

scale_x_continuous(breaks=seq(0,482,100)) +

scale_y_continuous(breaks=seq(0,250,50)) +

theme(panel.grid.major = element_blank(),

panel.grid.minor = element_blank(),

panel.background = element_blank(),

axis.line = element_line(colour = "black"),

axis.text.x = element_text(size = 20,hjust = 0.5,vjust = 0.5),

axis.text.y = element_text(size = 20,hjust = 0.5,vjust = 0.5),

axis.title.x = element_text(size = 23),

axis.title.y = element_text(size = 23),legend.position = "right")+

labs(x="",y="Follow up months")+

theme(axis.line = element_line(size=1, colour = "black"))+

geom_vline(aes(xintercept=241),colour="#BB0000",linetype="dashed")

p2

#3. p3是热图

#hubGene

library(pheatmap)

Group <- Group_DEG

Group$Group <- "High Score"

Group$Group[which(Group$LS ==1)] <- "Low Score"

hubgene_Exp <- as.data.frame(t(datExpr))

hubgene_Exphi <- hubgene_Exp[,which(colnames(hubgene_Exp) %in% GroupHigh)]

hubgene_Explo <- hubgene_Exp[,which(colnames(hubgene_Exp) %in% GroupLow)]

hubgene_Exp <- cbind(hubgene_Exphi,hubgene_Explo)

hubgene_Exp <- hubgene_Exp[which(rownames(hubgene_Exp) %in% hubgenes),]

bk <- c(seq(-2,0,by=0.01),seq(0.01,2,by=0.01)) #色度条调节

p3=pheatmap(hubgene_Exp,cluster_cols = F,cluster_rows = T,

show_colnames = F,show_rownames = T,

annotation_col = dplyr::select(Survival_group,5),scale = "row",

treeheight_row = 30,

color=c(colorRampPalette(colors=c("blue","white"))(length(bk)/2)

,colorRampPalette(color=c("white","red"))(length(bk)/2)),

legend_breaks = seq(-2,2,1),breaks = bk)

p3=ggplotify::as.ggplot(p3)

p3

#三图合并大法

library(cowplot)

plot_grid(p1,p2,ncol = 1,axis='l',align='v')

#####药物敏感性分析#####

library(data.table)

Drugsensitive <- fread('D:/AaPaper/Date整理/TCGA/LUAD/LUAD-WGCNA-Ferro药敏//DrugPredictions.csv', data.table = F)

rownames(Drugsensitive) <- Drugsensitive$V1

names(Drugsensitive)[1] <- 'sample'

#排序

Drug_high <- Drugsensitive[which(rownames(Drugsensitive) %in% GroupHigh),]

Drug_low <- Drugsensitive[which(rownames(Drugsensitive) %in% GroupLow),]

Drugsensitive <- rbind(Drug_high,Drug_low)

#多组小提琴图计算P值

pvalue <- as.data.frame(matrix(nrow = 198,ncol = 2))

names(pvalue) <- c("chemical","pvalue")

for (i in 2:199){

DrugtestHigh <- Drugsensitive[1:241,i]

DrugtestLow <- Drugsensitive[242:482,i]

wilcoxtest <- wilcox.test(DrugtestHigh,DrugtestLow)

p=round(wilcoxtest$p.value,3)

pvalue$chemical[i-1] <- names(Drugsensitive)[i]

pvalue$pvalue[i-1] <- wilcoxtest$p.value

}

pvalue$drug <- gsub("_.*$","",pvalue$chemical)

#分Pvalue

pvalue0.05 <- pvalue$chemical[which(pvalue$pval < 0.05 & pvalue$pval >= 0.01)]

pvalue0.01 <- pvalue$chemical[which(pvalue$pval < 0.01 & pvalue$pval >= 0.001)]

pvalue0.001 <- pvalue$chemical[which(pvalue$pval < 0.001)]

#分批

chemical1 <- Drugsensitive[,which(colnames(Drugsensitive) %in% pvalue0.05)]

colnames(chemical1) <- gsub("_.*$","",colnames(chemical1))

chemical2 <- Drugsensitive[,which(colnames(Drugsensitive) %in% pvalue0.01)]

colnames(chemical2) <- gsub("_.*$","",colnames(chemical2))

chemical3 <- Drugsensitive[,which(colnames(Drugsensitive) %in% pvalue0.001)]

colnames(chemical3) <- gsub("_.*$","",colnames(chemical3))

####假设rt：行是样本名字，列是细胞系

library(tidyr)

library(tidyverse)

#rt1 <- chemical1[,c(1,2,5,7,9,10,14,15,16,18,19,21)]

#0.1:c(4),1:c(3,6),60:c(1,2),200:c(7),300:c(),800:c(5,10)

#rt1 <- chemical2[,c(1,3,6,7,8,10,12,15)]

#rt1 <- chemical3[,c(1,2,3,4,7,8,10,11,12,13,14,16,24,25,27,34,35,36,37,39,43,44,47)]

#rt <- chemical3[,c(3,7,16,43)]#指南 IC60

rt <- cbind(chemical2[,1],chemical3[,c(1,13,14,34,35,47)])

#names(rt)[1] <- "Staurosporine"

rt$sample <- rownames(rt)

rt$Group <- "High"

rt$Group[which(rownames(rt) %in% GroupLow)] <- "Low"

rt <- rt %>% gather(key = chem,value = IC50,1:7)

library(ggpubr)

ggplot(rt,aes(x = Group,y = IC50,fill = Group))+

guides(fill = guide_legend(title = "Group"))+

labs(x = "", y = "IC50")+

geom_violin(alpha = 5,aes(linetype = NA))+

#scale_fill_manual(values = c("#E4C9DC","#61AEDC"))+ #填充颜色

facet_wrap(~chem,nrow =2)+theme_bw()+ylim(0,0.5)+

stat_compare_means(aes(group=Group),label = "p.signif",

method = "wilcox.test",hide.ns = T,cex =7.5,

hjust= 0,vjust = 1)+

geom_boxplot(width=0.2,cex=0.8,position=position_dodge(0.8),outlier.shape = NA)+

theme(axis.text.x = element_text(angle = 0, hjust = 0.5))

#####clinical characteristic#####

#单因素

library(survival)

library(survminer)

Survival_Clinical <- LUAD_Clinical[LUAD_Clinical$submitter_id.samples %in% rownames(datExpr),]

Survival_Clinical <- Survival_Clinical[,c(1,6,42,43,44,78,98)]

names(Survival_Clinical) <- c("sample","Age","M","N","T","Gender","Stage")

Survival_Clinical <- merge(Survival_Clinical,Survival_group,by = "sample")

Survival_Clinical$Stage <- gsub("stage ",'',Survival_Clinical$Stage)

Survival_Clinical$M <- gsub("NA","",Survival_Clinical$M)

Survival_Clinical$T <- gsub("b","",Survival_Clinical$T)

#####Cox回归~各变量中不显示的类型就是对照reference

##等级资料排序,cox回归的对照组就是等级最低的那一组

#factor(bb$Stage,ordered = F,levels = c(这里面从小到大排序))

Survival_Clinical$M <- factor(Survival_Clinical$M,ordered = F,levels = c("M0","M1","MX"))

Survival_Clinical$N <- factor(Survival_Clinical$N,ordered = F,levels = c("N0","N1","N2","N3"))

Survival_Clinical$T <- factor(Survival_Clinical$T,ordered = F,levels = c("T1","T2","T3","T4"))

Survival_Clinical$Gender <- factor(Survival_Clinical$Gender,ordered = F,levels = c("male","female"))

Survival_Clinical$Stage <- factor(Survival_Clinical$Stage,ordered = F,levels = c("I","II","III","IV"))

Survival_Clinical$group <- factor(Survival_Clinical$group,ordered = F,levels = c("Low Score","High Score"))

#Univariate (可信区间为0-infinite可能是对照组样本量太少)

library(survival)

characteritics <- Survival_Clinical

characteritics <- within(characteritics,{

Tstage <- NA

Tstage[T == "T1" | T == "T2"] = "T1/2"

Tstage[T == "M3" | T == "T4"] = "T3/4"

#Mstage[M == "M1" | M == "MX"] = "M1/X"

#Mstage[M == "M0"] = "M0"

#Nstage[N == "N1" | N == "N2" | N=="N3"] = "N1/2/3"

#Nstage[N=="N0"]="N0"

#stage[Stage == "I" | Stage == "II"] = "Stage I-II"

#stage[Stage == "III" | Stage == "IV"] = "Stage III-IV"

})

characteritics$T<-NULL

#循环函数构建所有变量

UNICOX<-function(x){

FML <- as.formula(paste0("Surv(OS.time,OS)~",x))

Clinical_cox <- coxph(FML, data=Survival_Clinical)

Clinical_sum <- summary(Clinical_cox)

Clinical_sum$coefficients

HR <- round(Clinical_sum$coefficients[,2],2)

pvalue <- round((Clinical_sum$coefficients[,5]),3)

CI <- paste0(round(Clinical_sum$conf.int[,3:4],2),collapse = "-")

unicox <- data.frame("characteristics"=x,

"Hazard Ratio"=HR,

"CI95"=CI,

"pvalue"=pvalue)

unicox$Variance<-rownames(unicox)

return(unicox)

}

UNICOX("Age")

library(plyr)

Clinical_var <- lapply(names(Survival_Clinical)[c(2:7,10)],UNICOX)

Clinical_var <- ldply(Clinical_var,data.frame)

Clinical_var #二分类变量的对照组需要从Sexsum里看，没有显示的那个是对照

write.csv(Clinical_var,file = "Plot/nomogram+calibration curve/univ.csv")

#多因素

fit_clinical <- coxph(Surv(OS.time,OS)~pred.multinom+Stage+Age+Gender,

x = T, y = T, data=Survival_Clinical)

sum_clinical <- summary(fit_clinical)

sum_clinical$coefficients

sum_clinical$conf.int

######subgroup#####

library(ggpubr)

subgroup <- LUAD_Clinical[LUAD_Clinical$submitter_id.samples %in% rownames(datExpr),]

subgroup <- subgroup[,c(1,6,42,43,44,78,98)]

names(subgroup) <- c("sample","Age","M","N","T","Gender","Stage")

subgroup <- merge(Survival_group,subgroup,by = "sample")

subgroup$Stage <- gsub("b",'',subgroup$Stage)

subgroup$M <- gsub("b","",subgroup$M)

subgroup$T <- gsub("a","",subgroup$T)

write.csv(subgroup,file = "clinical.csv")

subgroup2 <- read.csv("clinical.csv")

subgroup2$Stage[!is.na(subgroup2$Stage)] <- paste0("Stage",subgroup2$Stage[!is.na(subgroup2$Stage)])

subgroup2$Age65 <- "Age>62"

subgroup2$Age65[subgroup2$Age<63] <- "Age<=62"

ggviolin(subgroup2[!is.na(subgroup2$Gender),],x = "Gender",y = "pred.multinom",

color = "black",draw_quantiles = T,ylab = "Score",xlab = "",

alpha = 0.5,palette = "npg",size = 0.5,fill = "Gender",

add = c("boxplot")) +theme_classic2()+

stat_compare_means(method = "t.test",label = "p.signif",

hide.ns = T,size=6.5,vjust = 0.5,

comparisons = combn(unique(subgroup2[!is.na(subgroup2$Gender),]$Gender),2,simplify = F))+

#scale_x_discrete(limits = c("M0","M1","MX"))+

theme(text = element_text(size = 20))

#c("T1","T2","T3","T4")

#c("N0","N1","N2","N3")

#c("StageI","StageII","StageIII","StageIV")

library(survminer)

library(survival)

subgroup3 <- characteritics[characteritics$Tstage=="T3/4",]

subgroup3 <- subgroup[subgroup$M=="M1",]

fit_subgroup <- survfit(Surv(OS.time, OS) ~ group, data = subgroup3)

ggsurvplot(fit_subgroup, data = subgroup3, linetype = 1,

palette = c("#EE0000B2","#3B4992B2"),

size=1,surv.scale = c("percent"),pval = TRUE,legend.title = "M1",

legend.labs = c("High Scores", "Low Scores"),

break.time.by =12,

xlim = c(0,120),

risk.table = F,risk.table.title = "Patients at risk",

ylab = "Overall Survival, %",

xlab = "Months",font.x = c(20,"plain","black"),

font.y = c(20,"plain","black"),font.tickslab = c(18,"plain","black"),

risk.table.fontsize = 6.5,font.legend =c(20,"plain","black"),

font.main = c(20,"plain","black"),pval.size = 10)

####nomogram#####

library(regplot)

nomoclinical <- Survival_Clinical

nomoclinical$Stage <- factor(nomoclinical$Stage,ordered = F)

nomoclinical$N <- factor(nomoclinical$N,ordered = F)

nomoclinical$T <- factor(nomoclinical$T,ordered = F)

nomoclinical$M <- factor(nomoclinical$M,ordered = F)

nomoCox <- coxph(Surv(OS.time, OS) ~ pred.multinom +Stage,

data = Survival_Clinical)

summary(nomoCox)

regplot(nomoCox,failtime = c(12,36,60),observation = T,droplines=T,points = T,)

#####new model#####

library(survcomp)

pred.multinom2 <- as.data.frame(predict(f,nomoclinical))

rownames(pred.multinom2) <- nomoclinical$sample

names(pred.multinom2) <- c("pred")

pred.multinom2$sample <- rownames(pred.multinom2)

nomoclinical <- merge(nomoclinical,pred.multinom2,by="sample")

nomoclinical <- dplyr::arrange(nomoclinical,pred)

library(timeROC)

ROC_clinical <- timeROC(T = nomoclinical$OS.time,delta = nomoclinical$OS,

marker = nomoclinical$pred,weighting = "marginal",ROC = T,

times=c(12,24,36,48,60), cause = 1)

ROC_clinical

plot(ROC_clinical, time=12, col="#D86779", title=FALSE, lwd=2)

plot(ROC_clinical, time=24, col="#21A2A2",add=TRUE,title=FALSE, lwd=2)

plot(ROC_clinical, time=36, col="#E3B227", add=TRUE, title=FALSE, lwd=2)

legend(x=0.5, y=0.55,

c(paste0("AUC at 1 years: ", round (ROC_clinical$AUC[1],4)),

paste0("AUC at 2 years: ", round (ROC_clinical$AUC[2],4)),

paste0("AUC at 3 years: ", round (ROC_clinical$AUC[3],4))),

col=c("#D86779","#21A2A2","#E3B227") , lwd=2,bty="n")

cindexScore <- concordance.index(nomoclinical$pred.multinom[1:476],

surv.time = nomoclinical$OS.time[1:476],

surv.event = nomoclinical$OS[1:476],

method = "noether")

cindexClinical <- concordance.index(nomoclinical$pred,na.rm = T,

surv.time = nomoclinical$OS.time,surv.event = nomoclinical$OS,

method = "noether")

cindex.comp(cindexScore$data$surv.event,cindexClinical$data$surv.event)

######校准曲线#############

library(rms)

#Calculation of C-index

f <- coxph(Surv(OS.time,OS) ~ pred.multinom +Stage,

data = nomoclinical)

sum.surv<-summary(f)

sum.surv$concordance #cindex

sum.surv

c.index2<-t(as.data.frame(sum.surv$concordance))

c.index2

Low952 <- (c.index2[1]) - 1.96*(c.index2[2])

Upper952 <-(c.index2[1]) + 1.96*(c.index2[2])

c.index2<-cbind(c.index2[1], Low952, Upper952)

c.index2

#calibration curve

cal<- calibrate(coxm, cmethod = 'KM', method = 'boot', u = 60, m = 100, B = 100)

plot(cal,lwd=2,lty=1,errbar.col=c(rgb(0,118,192,maxColorValue=255)),

xlim=c(0.6,1), ylim=c(0.6,1), xlab='Nomogram-Predicted Probability of 5-Year OS', ylab='Actual 5-Year OS(proportion)', col=c(rgb(192,98,83,maxColorValue=255)))

lines(cal[,c('mean.predicted','KM')],type='b',lwd=2, col=c(rgb(192,98,83,maxColorValue=255)), pch=16)

abline(0,1,lty=3,lwd=2,col=c(rgb(0,118,192,maxColorValue=255)))

#calibration curve 1 year

f12<-cph(Surv(OS.time,OS) ~ pred.multinom +Stage,

x = T,y = T,surv = T,data = nomoclinical,time.inc = 12)

cal12<-calibrate(f12, cmethod="KM", method="boot",

u=12, #u需要与前面模型中time.inc一致，生存数据是月份，评估一年所以是12

m=100, #每次抽样的样本量，需要根据样本量确定，分成几组图中就显示几个点

B=1000) #抽样次数

par(mar=c(8,5,3,2),cex=1.0)

plot(cal12,lwd=2,lty=1,errbar.col=c(rgb(0,118,192,maxColorValue=255)),

xlim=c(0,1.0),ylim=c(0,1.0),

xlab="Nomogram-Predicted Probability of 1-year overall survival",

ylab="Actual 1-year overall survival (proportion)",

col=c(rgb(192,98,83,maxColorValue=255)))

#calibration curve 3 year

f36<-cph(Surv(OS.time,OS) ~ pred.multinom +Stage,

x = T,y = T,surv = T,data = nomoclinical,time.inc = 36)

cal36<-calibrate(f36, cmethod="KM", method="boot", u=36, m=100, B=1000)

par(mar=c(8,5,3,2),cex=1.0)

plot(cal36,lwd=2,lty=1,errbar.col=c(rgb(0,118,192,maxColorValue=255)),

xlim=c(0,1.0),ylim=c(0,1.0),

xlab="Nomogram-Predicted Probability of 3-year overall survival",

ylab="Actual 3-year overall survival (proportion)",

col=c(rgb(192,98,83,maxColorValue=255)))

#calibration curve 5 year

f60 <- cph(Surv(OS.time,OS) ~ pred.multinom +Stage,

x = T,y = T,surv = T,data = nomoclinical,time.inc = 60)

cal60 <- calibrate(f60, cmethod="KM", method="boot", u=60, m=100, B=1000)

par(mar=c(8,5,3,2),cex=1.0)

plot(cal60,lwd=2,lty=1,errbar.col=c(rgb(0,118,192,maxColorValue=255)),

xlim=c(0,1.0),ylim=c(0,1.0),

xlab="Nomogram-Predicted Probability of 5-year overall survival",

ylab="Actual 5-year overall survival (proportion)",

col=c(rgb(192,98,83,maxColorValue=255)))

######GSEA#####

LUADFerro <- LUAD_TPM[,colnames(LUAD_TPM) %in% rownames(datExpr)]

LUADFerro <- cbind(LUADFerro[,colnames(LUADFerro) %in% GroupHigh],

LUADFerro[,colnames(LUADFerro) %in% GroupLow])

write.csv(LUADFerro,file = "Plot/GSEA/LUADFerro.csv")
