# Supplementary figures and images for: Development of a ferroptosis-based model to predict prognosis, tumor microenvironment, and drug response for lung adenocarcinoma with weighted genes co-expression network analysis
Source: Front Pharmacol. 2022 Nov 17;13:1072589. doi: 10.3389/fphar.2022.1072589 (PMC9712758; doi:10.3389/fphar.2022.1072589)

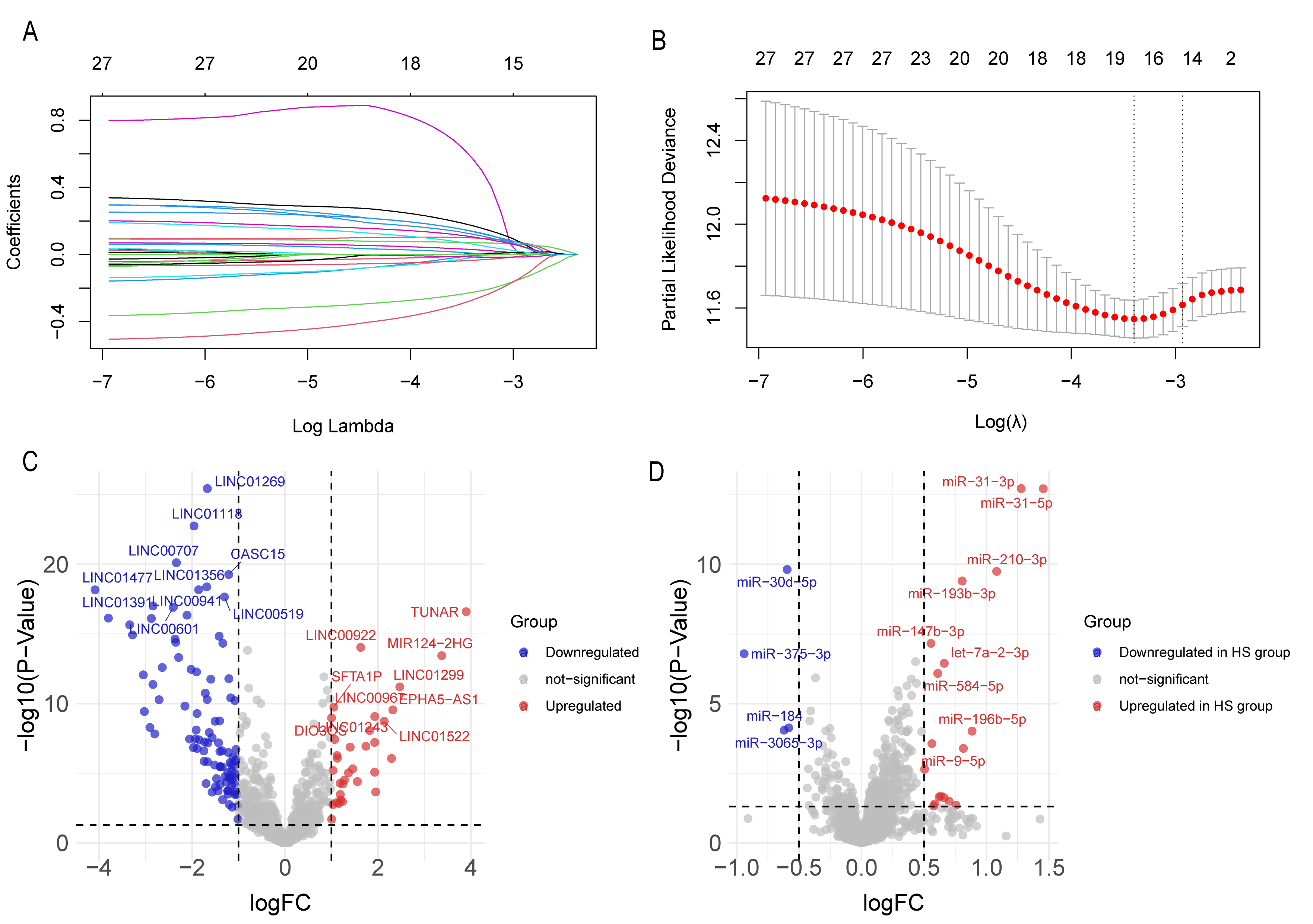

Supplement: Supplementary file 3 [file Image1.TIF]
